# Genome Mining of the Genus *Streptacidiphilus* for Biosynthetic and Biodegradation Potential

**DOI:** 10.3390/genes11101166

**Published:** 2020-10-03

**Authors:** Adeel Malik, Yu Ri Kim, Seung Bum Kim

**Affiliations:** 1 Department of Microbiology and Molecular Biology, Chungnam National University, Daejeon 34134, Korea; adeel@procarb.org (A.M.); aa432788@daum.net (Y.R.K.); 2 Institute of Intelligence Informatics Technology, Sangmyung University, Seoul 03016, Korea

**Keywords:** acidophile, *Streptacidiphilus*, *Streptomycetaceae*, *Kitasatospora*, *Streptomyces*, comparative genomics, specialized (secondary) metabolite, biosynthetic gene cluster, NRPS, PKS, CAZyme, biodegradation, pan-genome

## Abstract

The genus *Streptacidiphilus* represents a group of acidophilic actinobacteria within the family *Streptomycetaceae*, and currently encompasses 15 validly named species, which include five recent additions within the last two years. Considering the potential of the related genera within the family, namely *Streptomyces* and *Kitasatospora*, these relatively new members of the family can also be a promising source for novel secondary metabolites. At present, 15 genome data for 11 species from this genus are available, which can provide valuable information on their biology including the potential for metabolite production as well as enzymatic activities in comparison to the neighboring taxa. In this study, the genome sequences of 11 *Streptacidiphilus* species were subjected to the comparative analysis together with selected *Streptomyces* and *Kitasatospora* genomes. This study represents the first comprehensive comparative genomic analysis of the genus *Streptacidiphilus*. The results indicate that the genomes of *Streptacidiphilus* contained various secondary metabolite (SM) producing biosynthetic gene clusters (BGCs), some of them exclusively identified in *Streptacidiphilus* only. Several of these clusters may potentially code for SMs that may have a broad range of bioactivities, such as antibacterial, antifungal, antimalarial and antitumor activities. The biodegradation capabilities of *Streptacidiphilus* were also explored by investigating the hydrolytic enzymes for complex carbohydrates. Although all genomes were enriched with carbohydrate-active enzymes (CAZymes), their numbers in the genomes of some strains such as *Streptacidiphilus carbonis* NBRC 100919^T^ were higher as compared to well-known carbohydrate degrading organisms. These distinctive features of each *Streptacidiphilus* species make them interesting candidates for future studies with respect to their potential for SM production and enzymatic activities.

## 1. Introduction

The family *Streptomycetaceae* within the phylum *Actinobacteria* is one of the most diverse and ubiquitous soil bacterial groups [1] represented by three genera, classified as *Streptomyces*, *Kitasatospora* and *Streptacidiphilus*, which are closely related on phylogenetic and phenotypic aspects [2]. The representatives of this family are well-known for their roles in biodegradation (e.g., lignocellullose, chitin), secondary metabolite (SM) production and plant growth-promoting potential [3]. Among these three genera, *Streptacidiphilus* includes acidophilic species, and is phylogenetically more related with *Kitasatospora* as compared to *Streptomyces* [4,5]. They form branched substrate mycelia and aerial hyphae which are differentiated into long straight to flexuous chains of smooth surfaced spores. The whole-organism hydrolysates of *Streptacidiphilus* cell wall peptidoglycan contain LL-diaminopimelic acid (LL-DAP), a feature also observed in *Streptomyces* [5]. The cell wall peptidoglycan of *Kitasatospora*, on the other hand, contains either LL-DAP or *meso*-DAP in different parts of the mycelial structures [6].

Actinobacteria in general have been a great source of novel antibiotics, but the majority of these microbial compounds, such as cycloheximide [7], avermectins [8], hygromycin [9], and many others with known biological implications, have been derived from the family *Streptomycetaceae*, especially from *Streptomyces*. Similarly, the genus *Kitasatospora* has also been the producer of several important SMs, such as setamycin, phosalacine, and endophenaside antibiotics that are mainly active against eukaryotic organisms [10,11,12,13]. Representatives of *Streptomycetaceae* are also considered as the key organisms in carbon recycling because of their potential to degrade biopolymers [3]. However, only *Streptomyces* has been the focus of study in most of these studies [14,15]. Nevertheless, the potential of *Kitasatospora* in the degradation of biopolymers, chitosan in soil for example, has been demonstrated in a limited number of studies [16,17]. Recently, a group of tripeptides designated acidiphilamides were discovered as the first known SMs from a representative of *Streptacidiphilus*, namely *Streptacidiphilus rugosus* AM-16^T^ [18]. These acidiphilamides significantly inhibited cellular autophagy and highlighted the fact that under-investigated rare actinobacterial genera could serve as a good resource for novel bioactive natural products.

With the advancement in the genome sequencing technology, it was revealed that microorganisms still represent an essential source for novel natural products that need to be explored [19]. For example, the genome information of two well-studied species, *Streptomyces coelicolor* A3(2) [3] and *Streptomyces avermitilis* [20], revealed the presence of several cryptic biosynthetic gene clusters (BGCs), thereby highlighting their potential to produce new metabolites in addition to the existing ones from these organisms [21]. Since the complete genome sequence of *Str. coelicolor* A3(2) was first reported [3], a large number of genomes for this genus have become available in the public databases (e.g., https://www.ncbi.nlm.nih.gov/genome/). Similarly, the genomes of other two under-represented genera, viz, *Kitasatospora* and *Streptacidiphilus*, are also available in these databases, albeit to a limited extent. The genus *Streptacidiphilus* currently includes 15 validly named (http://www.bacterio.net/streptacidiphilus.html) acidophilic species, which in general grow in the pH range between 3.5 and 6.5, with optimum values around pH 4.5–5.5 [5].

Similar to *Streptomyces* and *Kitasatospora*, the distribution of *Streptacidiphilus* has also been reported from diverse terrestrial habitats such coal mine waste, acid mine drainage, and acidic forest soils [22,23]. Acidophilic actinobacteria are believed to be promising sources of compounds with antifungal activity and acid-stable enzymes [24,25]. Because soil acidophilic actinobacteria have to face competition with fungi in natural habitats, it is anticipated that they may have higher potential than their neutrophilic counterparts in some activities, e.g., antifungal activity [26,27]. Considering the rapid increase in the reports of new species in recent years, *Streptacidiphilus* may become a main source of novel metabolites together with the two neighboring genera.

Currently, 15 *Streptacidiphilus* genomes representing 11 species are available in public databases, which makes the genus the least studied one among the three genera of *Streptomycetaceae,* especially at the genome level. This is supported by the fact that no detailed genomic investigations have been carried out for this genus to date, although there are several brief reports for a limited number of *Streptacidiphilus* providing basic levels of analysis [4,28,29,30]. This study is a first comprehensive comparative genomic analysis of *Streptacidiphilus* to get a better understanding of their phylogeny, SM production and biodegradation capabilities. These *Streptacidiphilus* genomes were further compared with the representative *Kitasatospora* and *Streptomyces* species.

## 2. Materials and Methods

### 2.1. Streptacidiphilus Strains for Genome Analysis

Three representatives of *Streptacidiphilus*, *Streptacidiphilus albus* JL83^T^ (KCTC 9910^T^), *Streptacidiphilus rugosus* AM-16^T^ (KCTC 19279^T^) and *Streptacidiphilus oryzae* TH49^T^ (KCTC 19220^T^) were subjected to high quality genome sequencing, as these three species were described in earlier years and represented three different geographic locations. The biomass of the strains was obtained from the cultures grown at 30 °C for 3 days with shaking in ISP (International *Streptomyces* Project) medium 2 broth (glucose 0.4%, yeast extract 0.4%, malt extract 1%).

### 2.2. Genome Sequencing, Assembly and Annotation

A high quality genomic DNA for each strain was prepared using a DNA prep kit (Solgent, Daejeon, Korea), and DNA quantification was by PicoGreen dsDNA reagent kit (Thermo Fisher Scientific Korea, Seoul, Korea). Genome library construction, sequencing, and assembly were performed at the Joint Genome Institute (JGI, Berkeley, CA, USA) using PacBio RS [31] sequencing platform. The hierarchical genome assembly process (HGAP v2.2.0.p1) [32] was used to assemble the raw reads. Gene predictions were performed using Prodigal v2.5 [33] software. All coding sequences (CDS) were manually curated and verified by comparison with the publicly available NCBI database. Orthologous groups were assigned by using online functional annotation tool eggNOG-mapper (http://eggnog-mapper.embl.de/) [34], and also mapped to KEGG pathways by using the KEGG Automatic Annotation Server (KAAS: https://www.genome.jp/kegg/kaas/) [35]. Standalone antiSMASH v4.2.0 [36] with default parameters was used to predict the gene clusters that may have potential for the production of SMs. dbCAN2 (http://bcb.unl.edu/dbCAN2/) meta server [37] was used to annotate the carbohydrate active enzymes (CAZymes) [38]. Hits with an e-value threshold of ≤10^−5^ were considered only. Sequence similarity networks (SSN) were generated by using option C available at Enzyme Function Initiative-Enzyme Similarity Tool (EFI-EST: https://efi.igb.illinois.edu/efi-est/) [39] and visualized in Cytoscape v3.7.0 [40].

### 2.3. Comparative Genomic and Phylogenetic Analysis

For comparative analysis, the genomes of three *Streptacidiphilus* strains determined in this study, and an additional set of 8 *Streptacidiphilus* genomes retrieved from the NCBI Genome Database (https://www.ncbi.nlm.nih.gov/genome) were used. Only one genome of a type strain for each species was used in case there were two or more genomes for a given species. In addition, the genome data of 3 representatives each from *Kitasatospora* and *Streptomyces* were also retrieved from the NCBI Genome Database for comparative analysis. The partial 16S rRNA sequences of all *Streptacidiphilus* species were retrieved from public databases and aligned with ClustalW tool in the MEGA X [41] software. A neighbor-joining tree was constructed with a bootstrap test using 1000 resampled dataset, and evolutionary distances were computed by using the Jukes–Cantor method. Additionally, a whole genome based phylogeny was inferred using the Type (Strain) Genome Server (TYGS: https://tygs.dsmz.de/) webserver [42]. The average nucleotide identity (ANI) values across all seventeen (including 6 *Kitasatospora* and *Streptomyces*) genomes were calculated using orthoANIu [43]. The Bacterial Pan Genome Analysis (BPGA v1.3) [44] and bacterial pan-genome profile (PanGP v1.0.1) [45] pipelines were used to carry out the pan-genome analysis of all eleven *Streptacidiphilus* strains at 40% sequence identity cut-off.

## 3. Results and Discussion

### 3.1. General Features of Streptacidiphilus Genomes

The high-quality genome sequences of three *Streptacidiphilus* (*S. albus* JL83^T^, *S. rugosus* AM-16^T^, and *S. oryzae* TH49^T^) species were obtained for which the number of contigs ranged between 1 and 6. Among them, the genome of *S. albus* JL83^T^ was the largest (9.91 Mb) with a single contig. The DNA G+C content of this strain was 71.8 mol%, which is similar to *S. rugosus* AM-16^T^ of comparable genome size (9 Mb). The genome sequence of *S. rugosus* AM-16^T^ consisted of four contigs and the contig length of N50 was 7,115,445 bp. In contrast, the genome of *S. oryzae* TH49^T^ was smaller (7.81 Mb) with six contigs and N50 of 6,858,095 bp [28]. Besides these three *Streptacidiphilus* genomes, there are eight additional *Streptacidiphilus* strains for which genome data are available in the NCBI genome database. There were two entries for *S. albus* and *Streptacidiphilus jiangxiensis*, but only the entry with higher level of assembly was selected from each species. Among other *Streptacidiphilus* species retrieved from NCBI, only *Streptacidiphilus bronchialis* DSM 106435^T^ represented the high-quality genome, whereas the number of contigs/scaffolds for other genomes ranged between 96 and 281. The G+C content of all *Streptacidiphilus* strains was observed to be within the range of 70–72%, as described earlier [5], except *S. oryzae* TH49^T^ (strain with smallest genome size), in which the G+C content was observed as 73.4% and represented the highest among all these strains. Table 1 provides the summary of genomic features of all 11 *Streptacidiphilus* strains along with selected *Streptomyces* and *Kitasatospora* genomes. It can be noted that the number of pseudogenes is higher in both *Kitasatospora* and *Streptacidiphilus* than the *Streptomyces* genomes, although a higher number of pseudogenes has also been reported from *Streptomyces* as well [46]. Nevertheless, the function of these bacterial pseudogenes is debatable, and they are known to have short retention times as they undergo the process of degradation and removal by the accumulation of mutations [47].

**Figure 1:**
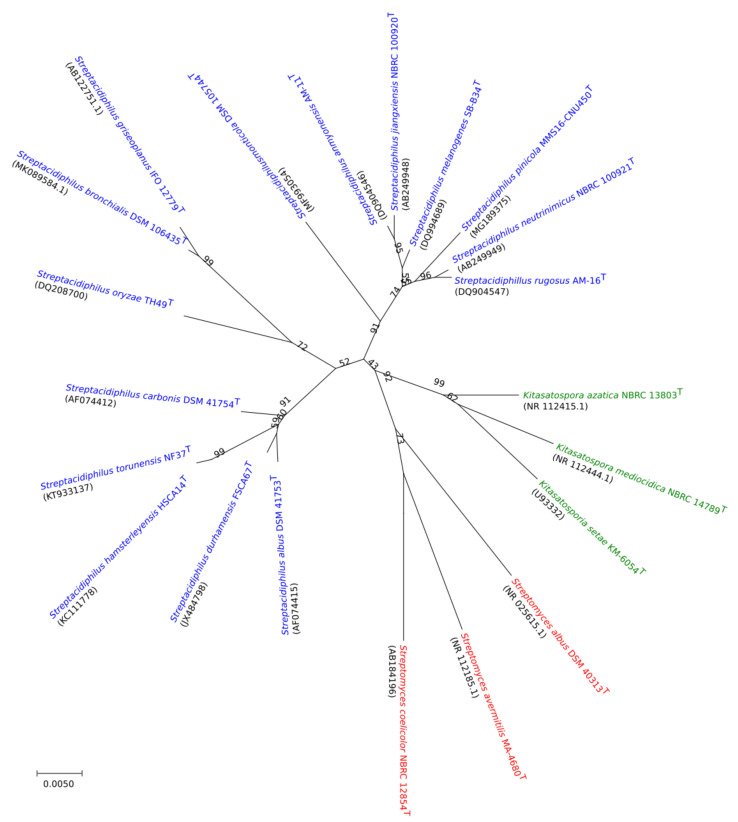
Neighbor-joining tree based on 16S rRNA gene sequences showing the relationships between 15 currently recognized species of *Streptacidiphilus*. Numbers at the nodes represent the bootstrap support (%), and the scale bar the substitutions rates per nucleotide position.

The orthoANIu values shared between the 11 *Streptacidiphilus* genomes ranged between 76.28% (*S. oryzae* TH49^T^ and *S. jiangxiensis* NBRC 100920^T^) and 91.14% (*Streptacidiphilus neutrinimicus* NBRC 100921^T^ and *Streptacidiphilus melanogenes* NBRC 103184^T^) (Table S1). *S. oryzae* TH49^T^ exhibited the least similarity (76.28 to 77.31%) with other representatives of *Streptacidiphilus*. The 16S rRNA gene sequences from all 15 validly described species were used as phylogenetic markers to confirm the taxonomic grouping into the genus *Streptacidiphilus* (Figure 1), in which each of the three genera were clearly separated from one another. Although the 16S rRNA-based phylogeny offers a handy method for deriving the relationships between species, it does not provide adequate taxonomic resolution for precise identification [48]. In contrast, the phylogenomic analysis of the strains used in this study and additional close neighbors of the query genomes predicted by TYGS webserver gave different views on the relationship among the three genera, as *Streptomyces* and *Streptacidiphilus* were paraphyletic while *Kitasatospora* remained monophyletic (Figure S1). In particular, *S. bronchialis* DSM 106435^T^ and *S. griseoplanus* IFO 12779^T^ as well as *S. oryzae* TH49^T^ fell outside the *Streptacidiphilus* group (Figure S1). The ANI indicated closer relationship of these species to *Kitasatospora*, but their reclassification does not seem straightforward as *S. oryzae* TH49^T^ was placed out of both *Streptacidiphilus* and *Kitasatospora* clades in the genome tree, and *S. bronchialis* DSM 106435^T^ and *S. griseoplanus* IFO 12779^T^ were reported to contain LL-diaminopimelic acid in the cell wall [49], which is not consistent with that of *Kitasatospora*. Further taxonomic studies would clarify the correct taxonomic affiliation of these taxa within the family.

### 3.2. Functional Annotation

**Figure 2:**
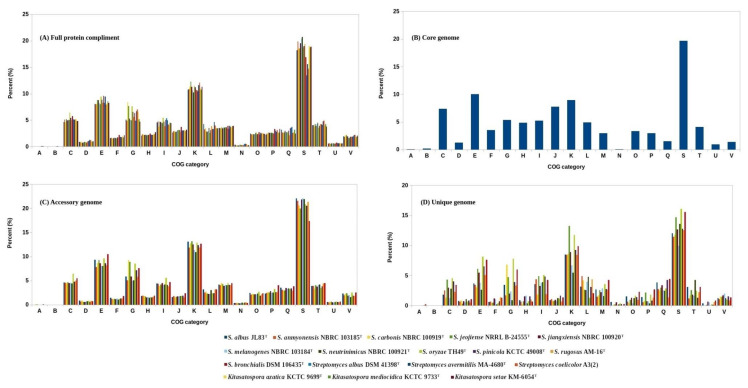

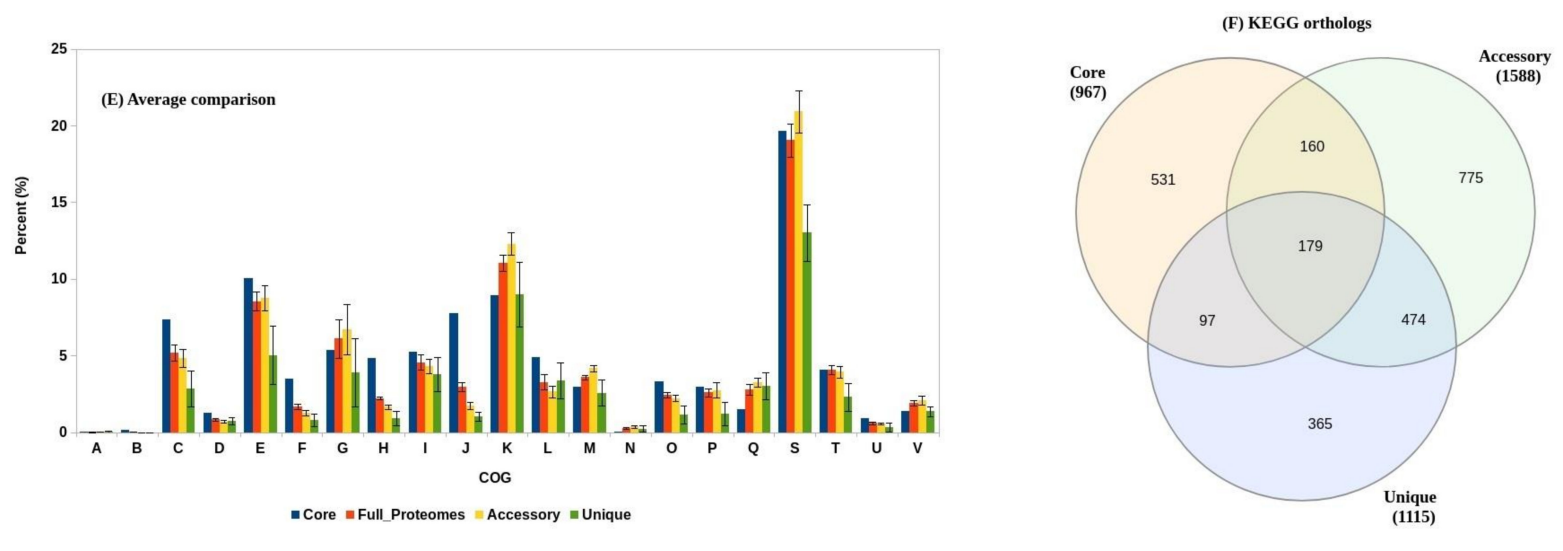
Distribution of COG categories in *Streptacidiphilus* genomes. (**A**) Full proteomes of *Streptacidiphilus* species, and for additional comparison representative strains from *Kitasatospora* and *Streptomyces* are added. (**B**) Core genome, (**C**) accessory genome, (**D**) unique genome, (**E**) average comparison, and (**F**) KEGG orthologs. The COG categories indicate RNA processing and modification (A), chromatin structure and dynamics (B), energy production and conversion (C), cell cycle control, cell division, chromosome partitioning (D), amino acid metabolism and transport (E), nucleotide metabolism and transport (F), carbohydrate metabolism and transport (G), coenzyme metabolism and transport (H), lipid metabolism and transport (I), translation, ribosomal structure and biogenesis (J), transcription (K), replication, recombination and repair (L), cell wall/membrane/envelop biogenesis (M), post-translational modification, protein turnover, chaperone functions (O), inorganic ion transport and metabolism (P), secondary metabolites biosynthesis, transport and catabolism (Q), function unknown (S), signal transduction mechanisms (T), intracellular trafficking, secretion, and vesicular transport (U), and defense mechanisms (V), respectively.

Assignment of orthologous groups and functional annotation revealed that on average about 11(±0.55)% of the proteins in each of the eleven *Streptacidiphilus* strains were assigned to the transcription (K) category, followed by 8.55(±0.59)% to amino acid transport and metabolism (E), and 6.12(±1.26)% to carbohydrate transport and metabolism (G) categories, respectively. Although all of these *Streptacidiphilus* strains exhibited more or less similar COG profiles, significant variation in the G category was observed. For example, the percentage of proteins annotated to carbohydrate transport and metabolism for *S. carbonis* NBRC 100919^T^ (8.37%), *Streptacidiphilus jeojiense* NRRL B-24555^T^ (7.66%) and *S. oryzae* TH49^T^ (7.59%) was much higher compared to other strains (Figure 2A). Overall, about 87% of the *S. oryzae* TH49^T^ and *S. bronchialis* DSM 106435^T^ proteins were observed to have orthologs in the COG database. For the remaining *Streptacidiphilus* strains, the percentage ranged between 80 (*S. albus* JL83^T^) and 86% (*S. jiangxiensis* NBRC 100920^T^). These figures include about 19% of the proteins in each genome belonging to unknown functional (S) categories. When the COG profile of these *Streptacidiphilus* strains was compared with the related representatives of *Streptomycetaceae*, both *Streptacidiphilus* and *Kitasatospora* were found to have higher number of proteins with unknown functions by about 5% than *Streptomyces*. This suggests that *Streptacidiphilus* along with *Kitasatospora* are the under-investigated representatives/genera of the family *Streptomycetaceae.*

Moreover, several enzymes belonging to various transposase families were also detected in each of the *Streptacidiphilus* genomes, especially in higher numbers in the high-quality (≤10 contigs) draft genomes. Studies have reported a highly variable number of transposase genes in prokaryotic genomes that can range from anywhere between 0 to several thousand per genome [58]. The most abundant transposase identified in the *Streptacidiphilus* genomes represented IS5/IS1182 family transposases. Transposases are mobile genetic elements and are believed to be crucial for the plasticity of bacterial genomes as well as host adaptations [59]. These transposases were also detected in the *Kitasatospora* and *Streptomyces* species used in this study. Similarly, 14, 7 and 7 CRISPR-associated (Cas) proteins were detected in the genomes of *S. albus* JL83^T^, *S. neutrinimicus* NBRC 100921^T^ and *S. bronchialis* DSM 106435^T^. Most of these Cas proteins were detected in the unique genomes of these three individual species, whereas no such proteins were annotated in other *Streptacidiphilus* genomes. The CRISPR-Cas systems have already been employed for the discovery and characterization of biosynthetic compounds from *Streptomyces* [60,61].

Large numbers of proteins from all strains were mapped to different types of metabolism-related KEGG pathways and exhibited a similar profile in all 17 strains. However, the top pathway in which the maximum number of proteins were mapped in all strains was “ABC transporter” pathway of KEGG database. Currently, the ABC transporter represents the largest protein family and is known to transport a wide range of molecules across the cellular membranes [62].

### 3.3. Pan-Genome Analysis

**Figure 3:**
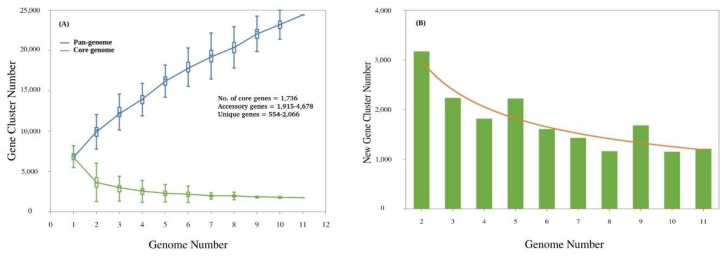
The pan-genome of *Streptacidiphilus* based on 11 species. (**A**) Core vs. pan developmental plot of eleven *Streptacidiphilus* genomes. The blue line indicates an increase in the number of genes in a pan-genome on sequential addition of genomes, and suggests an open pan-genome for this genus. In contrast, the number of core genes shared across these genomes decreases with the sequential addition of genomes, indicated by the red line. (**B**) Plot representing the number of new unique genes as a function of the number of strains added sequentially.

The pan-genome analysis of 11 *Streptacidiphilus* genomes resulted in 41,602 genes in the accessory/dispensable genome and 1736 sequences assigned to the core genome. The accessory genome of individual *Streptacidiphilus* strains ranged between 1915 (*S. bronchialis DSM 106435^T^*) and 4678 (*S. anmyonensis* NBRC 103185^T^) genes. Similarly, a total of 13,297 unique or genome-specific genes (singletons) were observed in *Streptacidiphilus* ranging between 554 (*S. melanogenes* NBRC 103184^T^) and 2066 (*S. albus* JL83^T^) genes. This pan-genome is slightly larger as compared to the pan-genome of 17 *Streptomyces* [63] and is expected to increase as the genomes of more *Streptacidiphilus* strains are sequenced. The *Streptacidiphilus* pan-genome shows the characteristics of an “open” pan-genome [64], the size of which increases with the sequential addition of new genomes (Figure 3A). The core genome profile also shows the expected gradual decrease with the sequential addition of new genomes. Similarly, the number of new genes does not converge to 0 as new genomes are added (Figure 3B). These observations are supported by the power law regression analysis, which shows that the pan-genome of *Streptacidiphilus* is in fact “open” with *Bpan* = 0.53. Such open pan-genomes are often found in bacterial species dwelling in diverse ecological habitats with complicated lifestyles, and show predisposition towards horizontal gene transfer (HGT) [64,65]. The *Streptacidiphilus* pan-genome was further discussed in terms of core, accessory and unique genes.

#### 3.3.1. Core Genome

The core genome of 11 *Streptacidiphilus* strains consisted of 1736 protein-coding genes (CDS). About 20% of these core genes were assigned to an unknown functional (S) category by COG analysis, whereas no orthologs were detected for approximately 4% of core CDS. Among the remaining core genome sequences, the enriched functional categories included amino acid metabolism and transport (E), transcription (K), translation, ribosomal structure and biogenesis (J), energy production and conversion (C), nucleotide metabolism and transport (F), carbohydrate metabolism and transport (G), and coenzyme metabolism and transport (H) (Figure 2B). Some of these categories in the core genome showed a higher percentage as compared to the average of full proteomes of each individual genome (Figure 2E). Coding sequences that belong to these orthologous groups are essential for basic cellular functions and survival [66], and also provides the major phenotypic traits [64]. The core genes were also mapped to about 191 KEGG pathways including ribosome, purine metabolism, and oxidative phosphorylation as the top three pathways with 52, 44 and 36 KEGG orthologs mapped to each of these pathways, respectively (Table S2A). Several other pathways implicated in amino acid and carbohydrate-related metabolism were also over-represented. These results are consistent with an earlier study on the related taxonomic group, *Streptomyces* [63], and highlight the fact that several core genes encoding transcriptional regulators and sigma factors may be a characteristic of this family and is in accordance with their sophisticated transcriptional regulatory system that impacts their morphological and physiological differentiation [67]. Overall, 967 unique KEGG orthologs were identified and assigned to 1105 coding sequences which represent about 64% of the core genome. This also means that some orthologs were assigned to more than one core gene. For example, at least 10 coding sequences from the core genome were mapped to KEGG ortholog “K01990”, an ABC-2 type transport system ATP-binding protein. In bacteria, such transporters aid the secretion of antibiotics through the cell membrane besides contributing towards self-resistance to the synthesized antibiotics [68].

#### 3.3.2. Accessory Genome

The accessory components of *Streptacidiphilus* genomes ranged between 1915 (*S. bronchialis* DSM 106435^T^) and 4678 (*S. anmyonensis* NBRC 103185^T^) CDS (Table 1). On average, a slightly higher percentage (~21%) of accessory genomes for each *Streptacidiphilus* strain were assigned to an unknown functional category (S). In fact, this percentage is highest among the core, full as well as the unique genomes (Figure 2E), and this may suggest that certain specific functional roles of *Streptacidiphilus* are probably performed by these accessory genes, which nevertheless remains to be established experimentally. In addition, a slightly higher number of accessory CDS were assigned to K, G, cell wall/membrane/envelop biogenesis (M), and secondary metabolites biosynthesis, transport and catabolism (Q) categories as compared to the CDS of other genomes (Figure 2C). Generally, the accessory genome offers diversity within a species, and may perform functions and pathways that are secondary for bacterial growth but may be otherwise advantageous to overcome adverse environmental conditions [64]. Furthermore, the accessory genome was mapped to 273 (higher than the core genome) KEGG pathways with 119, 74 and 42 orthologs detected for ABC transporters, two-component system, and amino sugar and nucleotide sugar metabolism pathways, respectively (Table S2B). About 13,376 (32%) accessory sequences were mapped to 1588 KEGG orthologs. The topmost ortholog, to which 249 sequences from the representative accessory genome were mapped, was “K00059” with 3-oxoacyl-[acyl-carrier protein] reductase (fabG, OAR1) activity. In addition to their roles in fatty acid metabolism, these reductases are also involved in the biosynthesis of prodigiosin and antibiotics (https://www.genome.jp/dbget-bin/www_bget?ko:K00059). Such enzymes are also attractive targets for the design of new antimicrobial compounds [69]. The second most abundant KEGG ortholog found in the accessory genome was again ABC-2 type transport system ATP-binding protein (“K01990”), to which sequences were mapped. The other ortholog to which as many as 149 accessory sequences were mapped was “K12132”, which is a eukaryotic-like serine/threonine-protein kinase (prkC, stkP). Studies have shown that such kinases are present in several prokaryotes and regulate key processes related to cell division, morphogenesis and development, although their substrates and mechanisms of action vary from one bacterial species to another [70]. The large number of stkP orthologs observed in the accessory genome of *Streptacidiphilus* could be attributed to the fact that several eukaryotic-like serine/threonine-protein kinase (ESTPK) encoding genes have been reported from related *Streptomyces*. For example, the genome sequence of *Str. coelicolor* A3(2) led to the identification of at least 34 putative ESTPK genes [71].

#### 3.3.3. Unique Genome

A maximum of 2066 singletons were observed in *S. albus* JL83^T^, whereas *S. melanogenes* NBRC 103184^T^ contained only 554 singletons (Table 1). Comparison of the species-specific COG profiles highlighted the presence of a large number of singletons from *S. oryzae* TH49^T^ annotated for carbohydrate transport and metabolism (G) category (Figure 2D), which included those for several proteins with CAZyme domains.

Similar to the accessory genome, the average number of CDS annotated in Q category of COG was slightly higher in all strain-specific genes as compared to the core and full genome CDS (Figure 2E). The number of strain-specific NRPS proteins ranged between 1 (*S. neutrinimicus* NBRC 100921^T^) and 9 (*S. bronchialis* DSM 106435^T^). Similarly, at least 18 PKS singletons were detected in *S. bronchialis* DSM 106435^T^ genome, which is almost three times the number of PKS singletons identified in the genomes of *S. albus* JL83^T^ and *S. anmyonensis* NBRC 103185^T^. Moreover, strains such as *S. carbonis* NBRC 100919^T^, *S. jeojiense* NRRL B-24555^T^, and *S. oryzae* TH49^T^, which have shown a higher number of proteins annotated in G category, exhibited no or a limited number of NRPS and PKS proteins in their unique genomes. The top two KEGG pathways detected in the unique genomes were similar to those of accessory genomes (i.e., ABC transporters, two-component system). However, the third top KEGG pathway identified within the unique genomes was pyruvate metabolism (Table S2C). Additionally, the most abundant KEGG orthologs with a maximum of 62 and 46 orthologs were “K00059” and “K12132”, respectively, as described above for the accessory genomes.

Overall, there were about 179 KEGG orthologs shared commonly between core, accessory and unique genomes (Figure 2F). Individually, the number of orthologs mapped to the unique genomes was much lower (365) as compared to the orthologs identified for core (531) and accessory (775) genomes. These data are consistent with the COG analysis in which the unique genome was least annotated (56.56%, ±17.77) as compared to other genomes, thereby again suggesting the potential novel/unique functions carried out by the CDS of unique genomes.

### 3.4. Genes Related to Morphological Properties

Considering the fact that *Streptacidiphilus* shares key chemotaxonomic and morphological properties with related *Streptomyces* and *Kitasatospora* [3], the genes involved in the development of aerial mycelium and cell wall peptidoglycan were investigated. Several orthologs of the proteins encoded by *bld* cascade genes such as *bldA*, *bldB*, *bldC*, *bldD*, *bldG*, *bldH*, *bldKA*, *bldM*, *bldN*, and *amfC* that are involved in the formation of aerial mycelium [72,73] were observed in the genomes of all *Streptacidiphilus* strains. However, some of these genes were not identified straightforwardly and required local BLAST search. For example, the amino acid sequence (AAA79120) of *bldB* gene from *Str. coelicolor* A3(2) was used as a query and scanned against the proteomes of individual *Streptacidiphilus* species, and exhibited about 40–55% sequence identity with the potential *bldB* orthologs from all *Streptacidiphilus* strains. All *bldB* protein orthologs including the amino acid sequence from *Str. coelicolor* A3(2) were assigned to the category of unknown function (DUF397: http://pfam.xfam.org/family/PF04149) in the Pfam [74] database. In contrast, the amino acid sequence (NP_627022) encoded by *bldH* (also known as *AdpA* homologue) gene from *Str. coelicolor* A3(2) exhibited about 80-86% sequence identity with the potential *bldH* protein sequences of almost all strains. The only exception to this higher sequence identity was observed in the cases of *S. albus* JL83^T^, *S. carbonis* NBRC 100919^T^ and *S. melanogenes* NBRC 103184^T^, in which the amino acid sequence identity for this gene was observed to be 49.37 (WP_034091671), 50.15 (WP_042407209) and 48.23% (WP_042383536), respectively. The *bldH* protein regulated by *bldA* is an essential intermediate transcription regulator in the *bld* cascade [75]. Similar to *bld* developmental master regulators, several *whi* family genes (e.g., *whiA*, *whiB*, *whiG*, *whiH*) that are involved in regulating sporulation [73] were also identified in all *Streptacidiphilus* genomes. Mutational studies have shown that *bld* mutants fail to develop aerial hyphae and lack the typical “fuzzy” appearance of the wild type, therefore showing a shiny, “bald” phenotype. In contrast, *whi* mutants are capable of forming aerial hyphae, but cannot complete their life cycle by forming mature spore chains [76,77].

The cell wall peptidoglycan of *Streptacidiphilus* resembles that of *Streptomyces* and also contains LL-diaminopimelic acid (LL-DAP) as the major diamino acid [5]. The *murE* genes encode enzymes that catalyze the formation of the UDP-*N*-acetylmuramic acid (UDP-MurNAc) tripeptide in the biosynthesis of bacterial peptidoglycan [78]. Two *murE* genes in the genomes of all three *Streptacidiphilus* species were identified, which is akin to most of the *Streptomyces* and *Kitasatospora* genomes. The first *murE* gene encodes a protein with UDP-*N*-acetylmuramoyl-L-alanyl-D-glutamate-2,6-diaminopimelate ligase activity, and the length of this enzyme ranged between 500 and 580 amino acid residues in 11 *Streptacidiphilus* strains as well as in *Streptomyces* and *Kitasatospora*. However, an additional homologue (490 amino acids) of this first *murE* gene was identified in *Kitasatospora azatica* KCTC 9699^T^ and can be seen as an outlier in Figure S2A. In contrast, the second *murE* gene encodes a shorter protein with approximately 400 amino acids in *Streptacidiphilus* and other strains of *Streptomycetaceae*. This shorter murE protein contains a domain of an unknown function (DUF1727) which is present towards the C terminal region of bacterial proteins that include UDP-*N*-acetylmuramyl tripeptide synthase and the related Mur ligase (http://pfam.xfam.org/family/PF08353.10). On the other hand, only one copy of *dapF* gene, the product of which is involved in the isomerization of LL-DAP into *meso*-DAP [79,80] was identified in most *Streptacidiphilus* genomes, except for *Streptacidiphilus anmyonensis* NBRC 103185^T^ and *S. neutrinimicus* NBRC 100921^T^, in which two copies of this gene were present in both genomes (Figure S2B). This is consistent with earlier studies where the occurrence of more than one *dapF* genes was reported in some species of *Kitasatospora* and *Streptomyces* [81].

### 3.5. Predicted BGCs of Streptacidiphilus

Table 1 gives the overview of the number of BGCs identified in each genome. Overall, 506 BGCs were predicted in the 17 genomes used in this study, including 305 from 11 *Strepacidiphilus*. The number of BGCs for *Streptacidiphilus* ranged between 15 (*S. oryzae* TH49^T^) and 38 (*S. neutrinimicus* NBRC 100921^T^), which corresponds to 1.92 and 4.15 BGCs per Mb, respectively. The average number of BGCs per Mb was 3.17(±0.85).

The distribution of most abundant types of BGCs for SMs detected in each *Streptacidiphilus* genome is summarized in Table 2. From the table, it can be seen that the BGCs for siderophores and terpenes are the only clusters that are commonly present in all 11 *Streptacidiphilus* genomes as well as representative *Kitasatospora* and *Streptomyces*. The number of non-ribosomal peptide synthetase (NRPS) gene clusters in *Streptacidiphilus* ranged between 1 (*S. bronchialis* DSM 106435^T^ and *S. melanogenes* NBRC 103184^T^) and 5 (*S. jiangxiensis* NBRC 100920^T^). A few NRPS clusters were also present as a part of hybrid clusters, whereas no NRPS cluster (including hybrids) was observed in *S. oryzae* TH49^T^. None of the *Streptacidiphilus* strains with high quality genomes (≤10 contigs) possessed type 1 polyketide synthase (T1PKS) gene clusters, except as a component of hybrid clusters. A broad variety of potential structures predicted from these BGCs are shown in Table S3. Some of the important types of BGCs that may be involved in the synthesis of these potential compounds are discussed below.

Since 7 out of 11 *Streptacidiphilus* genomes have several contigs, it is expected to have a number of BGCs to be located at contig edges, giving some level of redundancy in the number of BGCs. For example, the number of BGCs predicted at contig edges in draft *Streptacidiphilus* genomes (genomes with number of contigs between 96–281) ranged between 4 in *S. jeojiense* NRRL B-24555^T^ (144 contigs) and 22 in *S. neutrinimicus* NBRC 100921^T^ (184 contigs). In contrast, only two (one each in *S. albus* JL83^T^ and *S. oryzae* TH49^T^) BGCs were identified in strains with high-quality (≤10 contigs) genomes. Similarly, only one BGC in the case of *Str. albus* DSM 41398^T^ was detected on the contig edge among all the representative *Streptomyces* and *Kitasatospora* genomes. Although the number of BGCs from draft genomes may be overpredicted, they would offer a worthy initial measure of the potential biosynthetic diversity [82].

#### 3.5.1. Type 1 Polyketide Synthase (T1PKS) BGCs

The T1PKS with 36 such BGCs distributed among 11 *Streptacidiphilus* species was one of the highly abundant types of SM producing gene clusters. However, as mentioned above, none of the four high-quality *Streptacidiphilus* genomes contained T1PKS clusters except as a part of hybrid clusters. Of all the T1PKS clusters, 11 were detected in *S. neutrinimicus* NBRC 100921^T^, but most of these clusters were small and consisted of only one to five genes, except in the case of clusters 9, 14, 27 and 31. Similarly, most of the T1PKS type BGCs from *S. carbonis* NBRC 100919^T^ consisted of a few genes only, except for cluster 15, which consisted of several PKS enzymes with well-defined modular structure and exhibited 30% similar genes with geldanamycin BGC from *Streptomyces hygroscopicus* NRRL 3602 in MIBiG (https://mibig.secondarymetabolites.org/) [83,84] database. In contrast, five out of the six BGCs from *S. melanogenes* NBRC 103184^T^ consisted of multiple genes with well-defined modular structures of their PKS enzymes. Among the T1PKS clusters from *S. melanogenes* NBRC 103184^T^, cluster 4 exhibited a maximum of 39% of similar genes with ECO-02301 BGC from *Streptomyces aizunensis*, the compound known as an antifungal agent [85]. The low similarities with the known BGCs suggest that the potential T1PKS biosynthetic compounds predicted by antiSMASH from *Streptacidiphilus* species (Table S3) may be novel. Some of these BGCs did not show any similarity with the known BGCs available in the databases. Overall, among all the T1PKS BGCs, cluster 17 from *S. anmyonensis* NBRC 103185^T^ shared the maximum gene content similarity (60% similar genes) with the known ebelactone A BGC from *Kitasatospora aburaviensis* [86].

#### 3.5.2. Non-Ribosomal Peptide Synthetase (NRPS) BGCs

Overall, 28 NRPS BGCs were identified from 11 *Streptacidiphilus* strains, which include 7 clusters from 4 (*S. albus* JL83^T^, *S. oryzae* TH49^T^, *S. rugosus* AM-16^T^ and *S. bronchialis* DSM 106435^T^) high quality draft genomes. Among these, four NRPS clusters were detected from *S. albus* JL83^T^, three (clusters 9, 26 and 30) out of which consisted of NRPS proteins with well-defined modular structures (Figure S3). Although a core structure was predicted for all 4 NRPS clusters (Table S3), the gene content similarities with existing BGCs were significantly low (<25%), except for cluster 30, in which 50% of genes showed similarity with cyanopeptin BGC from *Planktothrix agardhii*, a filamentous cyanobacterium [87]. However, the amino acid identities between the individual NRPS sequences from *P. agardhii* and *S. albus* JL83^T^ were around 40%, in addition to the varying modular structures and domain organization. In the case of *S. rugosus* AM16^T^, only cluster 18 consisted of NRPS proteins, having a well-defined modular structure. Only 28% of genes from this cluster exhibited similarity with a known BGC for erythrochelin, a siderophore. A single NRPS cluster (cluster 17) was detected in the genome of strain *S. bronchialis* DSM 106435^T^, which shared only 8% gene content similarity with the BGC for cyclomarin, an antimycobacterial and antimalarial cyclopeptide.

Overall, each of these abovementioned NRPS clusters ranged in size approximately between 58 (cluster 9 of *S. albus* JL83^T^) and 82 Kbp (cluster 17 of *S. bronchialis* DSM 106435^T^). A high degree of variation in the type, number and organization of various NRPS domains among all these clusters was also observed (Figure S3). For example, in addition to the varying number of classical condensation (C), adenylation (A) and thiolation (T, also known as peptidyl carrier protein (PCP) domain) domains, certain tailoring domains such as epimerisation (E) or β-ketoreductase (KR) domains were also present in some strains. Additionally, each of these clusters also included genes responsible for transcription regulation and transport.

Similarly, those of the other seven *Streptacidiphilus* strains (>90 contigs) also exhibited low gene content similarities with the known BGCs except in the case of cluster 8 from *S. jeojiense* NRRL B-24555^T^ that showed 100% similarity with scabichelin BGC from *Streptomyces scabiei* 87.22 [88]. The scabichelin BGC contains a core gene that encodes a putative NRPS/siderophore biosynthesis protein in addition to other transport-related genes. These putative NRPS proteins from *S. jeojiense* NRRL B-24555^T^ and *Streptomyces scabiei* 87.22 show very similar domain architecture with five C-A-T domains and a single E domain, and exhibited about 70% sequence identity over a coverage of 45%. In spite of such similarities, a difference in the overall predicted structures from these two clusters was observed (Figure S4A). One of the main differences between these two species is that in the case of *Streptomyces scabiei* 87.22, a second central n-methylation (nMT) domain as well as an additional domain of about 135 amino acid residues is also found towards the C-terminal end. The scabichelin BGC was also predicted in the genomes of *K. azatica* KCTC 9699^T^ and *Kitasatospora setae* KM-6054^T^, although exhibiting only 20% gene content similarity to the known scabichelin BGC, and the putative NRPS/siderophore biosynthesis proteins in both these cases were smaller in length with no predicted domain organization.

#### 3.5.3. T1PKS-NRPS Hybrid Gene Clusters

About 51 hybrid BGCs were detected from the 10 *Streptacidiphilus* genomes, 17 of which were found in *S. albus* JL83^T^. These hybrid clusters were formed by the combination of two or more different types of BGCs and could be as simple as commonly observed T1PKS-NRPS hybrids or as complex as butyrolactone-T2PKS-terpene-siderophore BGC observed in *S. jiangxiensis* NBRC 100920^T^. At least one such hybrid cluster was observed in all *Streptacidiphilus* strains except *S. oryzae* TH49^T^. The most abundant type of hybrid cluster was T1PKS-NRPS, and there were seven such clusters from *S. bronchialis* DSM 106435^T^ (cluster 9, 14 and 15), *S. albus* JL83^T^ (cluster 5), *S. carbonis* NBRC 100919^T^ (cluster 14), *S. jiangxiensis* NBRC 100920^T^ (cluster 26) and *S. neutrinimicus* NBRC 100921^T^ (cluster 23). The three T1PKS-NRPS clusters from *S. bronchialis* DSM 106435^T^ shared 10%, 64% and 50% gene content similarities with thioviridamide, neocarzilin and abyssomicin BGCs, respectively. Although thioviridamide is synthesized by ribosomally synthesized and post-translationally modified peptide (RiPP) family BGC [89], there are some common genes present in T1PKS-NRPS (cluster 9) and the known thioviridamide BGC from *Streptomyces olivoviridis* [89]. For example, LuxR family transcriptional regulators which are involved in quorum-sensing (QS) mechanisms [90] were detected in both BGCs. Thioviridamide and neocarzilin are known to exhibit antitumor activities, and abyssomicin to exhibit antibacterial activities. In contrast, the lone T1PKS-NRPS hybrid cluster from *S. albus* JL83^T^ consisted of genes that were 100% similar with the BGC of antimycin, a fish poison from *Streptomyces* sp. S4 [91]. The gene content similarity between the NRPS sequence of both these organisms was 60%, whereas PKS sequences exhibited 66% sequence identity with ~100% coverage. In addition to high amino acid sequence similarities observed between NRPS/PKS sequences of these two strains, their modular organizations were also very similar (Figure S4B). However, one of the main differences between these two clusters was that the T1PKS-NRPS cluster in *S. albus* JL83^T^ genome was much bigger (~135 Kbp), almost twice the size of antimycin BGC of *Streptomyces* sp. S4, and consisted of additional genes including two NRPS, one PKS, and other tailoring genes. Among the additional NRPS genes, one showed 57% gene content similarity with the NRPS protein sequence (WP_067134481) from *Microtetraspora malaysiensis*, whereas the other NRPS sequence shared 52% amino acid sequence identity with adenylation domain-containing protein (WP_123974827) from *Streptomyces* sp. Ag109_O5-1. In contrast, the amino acid sequence of additional PKS exhibited 54% identity with T1PKS (WP_095581742) from *Streptomyces albireticuli*. This cluster in fact was also probably the largest T1PKS-NRPS cluster as compared to three other such hybrid clusters from *S. bronchialis* DSM 106435^T^ that ranged between 76 (cluster 9) and 111 Kbp (cluster 15). Therefore, these data indicate that in spite of high gene content similarities shared between the T1PKS-NRPS cluster (cluster 5) from *S. albus* JL83^T^ and antimycin BGC from *Streptomyces* sp. S4, the potential SM produced by *S. albus* JL83^T^ from this cluster could be novel.

In contrast, cluster 14 of *S. carbonis* NBRC 100919^T^ showed 24% gene content similarity with the himastatin (BGC0001117) BGC, whereas only 10% of the genes from cluster 23 of *S. neutrinimicus* NBRC 100921^T^ exhibited similarity with the enduracidin (BGC0000341) BGC of MIBiG database.

#### 3.5.4. Other Hybrid Gene Clusters

In addition to T1PKS-NRPS clusters, the other hybrid clusters either involved a PKS or an NRPS cluster in combination with other types (bacteriocin-T2PKS, butyrolactone-T1PKS-otherks, T2PKS-oligosaccharide-otherKS, terpene-butyrolactone etc). A total of 33 such hybrid clusters were detected, 9 of which belonging to *S. albus* JL83^T^, whereas no hybrid cluster was detected in *S. oryzae* TH49^T^ (Table 2 and Table S2). Some of these clusters from *S. albus* JL83^T^ contained well-defined modular structures of PKS or NRPS domains, for example clusters 7 (Terpene-T1PKSs), 20 (T3PKS-NRPS) and 22 (T3PKS-lanthipeptide-otherks-NRPS) while exhibiting limited gene content similarities (between 21–42%) with known BGCs. In contrast, 51% genes of T3PKS-T1PKS hybrid cluster (cluster 16) from strain DSM 106435 showed similarity with the known BGCs for PM100117/PM100118, which are antitumor substances from *Streptomyces caniferus* [92].

#### 3.5.5. Lanthipeptide Gene Clusters

At least 28 (including hybrids) lanthipeptide BGCs were identified from 11 *Streptacidiphilus* strains, 7 out of which were present in *S. albus* JL83^T^. Based on antiSMASH analysis, the seven lanthipeptide clusters from *S. albus* JL83^T^ had potential for class I (clusters 10, 21 and 23) or class II (clusters 6 and 24) lanthipeptides. In addition to these seven lanthipeptide clusters, one T3PKS-lanthipeptide-otherks-NRPS hybrid cluster (cluster 22) was also observed in *S. albus* JL83^T^. While only one lanthipeptide was predicted for most clusters, multiple class II core peptides were predicted for clusters 22 and 24 (Table S4). No core peptides were predicted for clusters 13 and 33. In contrast, three lanthipeptide BGCs (clusters 8, 10 and 12) from *S. bronchialis* DSM 106435^T^ were classified as those for class I lanthipeptides and predicted to have a core peptide associated with each cluster. Similarly, a core lanthipeptide belonging to class I was predicted for *S. carbonis* NBRC 100919^T^ (cluster 22), *S. jeojiense* NRRL B-24555^T^ (cluster 18), and *S. jiangxiensis* NBRC 100920^T^ (cluster 15). Apart from *S. albus* JL83^T^, *S. anmyonensis* NBRC 103185^T^ (cluster 9: hybrid lanthipeptide-T1PKS)*, S. melanogenes* NBRC 103184^T^ (cluster 8: hybrid T2PKS-lanthipeptide)*,* and *Streptacidiphilus pinicola* KCTC 49008^T^ (cluster 27) were also predicted to have two, three, and two class II core peptides, respectively (Table S4). Class I lanthipeptide clusters contain a lanthipeptide dehydrogenase (LanB) and a cyclase (LanC), both of which are required for the enzymatic synthesis of lanthipeptides. Class II lanthipeptides, on the other hand, are altered by a bifunctional “LanM” enzyme which comprises an N-terminal dehydratase domain and a C-terminal LanC-like cyclase domain [93]. Although all class I and class II lanthipeptides from 11 *Streptacidiphilus* genomes contained essential core enzymes for the biosynthesis of lanthipeptides, the number and types of tailoring enzymes varied, which may offer additional modifications specific for each lanthipeptide and ameliorate their activities and/or stability [94].

#### 3.5.6. Terpene Gene Clusters

The biosynthesis of terpenes is in general investigated by determining the products of an individual enzyme known as terpene synthase or commonly referred to as terpene cyclase [95]. However, the synthesized compound may require additional modifications by the action of additional genes to attain biological activity. Therefore, it is essential to identify the underlying gene clusters, which in turn is necessary for the discovery of unique metabolic pathways and their potential industrial applications [96]. In *Streptacidiphilus*, a total of 53 terpene BGCs were identified, which were found as the most abundant type of BGCs in this genus. *S. oryzae* TH49^T^, with eight such clusters, was the top strain, followed by six each for *S. jiangxiensis* NBRC 100920^T^ and *S. carbonis* NBRC 100919^T^. These terpene clusters shared limited similarities with the known BGCs, and hopene was one of the most common matches. In addition to core biosynthetic terpene/phytoene synthases/cyclases, some clusters contained additional biosynthetic enzymes such as phytoene desaturases, dehydrogenases, and glycosyl transferases, etc. Several regulatory and transport genes were also observed in these clusters. Such genes are required for the biosynthesis of terpenes [97], but the number and types of these genes varied across all terpene BGCs. Although knowledge on the terpene production from bacteria is limited, some terpene or terpenoid structures have been reported in addition to few characterized biosynthetic pathways [98]. For example, phenalinolactone (a terpene glycoside), produced by *Streptomyces* sp. Tü6071 [79], is encoded by a 35-gene cluster and comprises all the biosynthetic as well as regulatory genes required for its production. Similarly, terpenes such as terpenticin and brasilicardin A from other bacterial species are also produced by BGCs with partially characterized biosynthetic pathways [99,100].

#### 3.5.7. Distribution of Known BGCs in Streptomycetaceae

**Figure 4:**
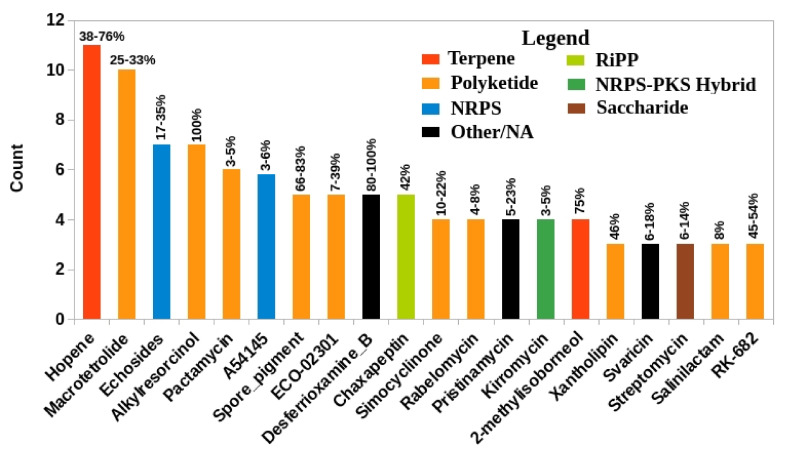
Most abundant known BGCs in *Streptacidiphilus*. Bar color indicates the classification of each cluster type in MIBiG database. Numbers above the bars indicate the range of percent similarity shown the clusters against known BGCs.

As mentioned above, terpene is one of the most abundant BGCs found in the *Streptacidiphilus* strains, and the number still remains higher and reaches 78 if the representatives from *Streptomyces* and *Kitasatospora* are included (Table 2). The most abundant known BGC present in all these strains was hopene (Figure 4), a terpene-type BGC. Each of the 17 species used in this study consisted of 1 hopene BGC and showed a similarity within a range of 38 (some *Streptacidiphilus* strains) to 100% (*Str. coelicolor*) with the reference hopene BGC (BGC0000663) in the MIBiG database. Hopenes play roles in maintaining membrane fluidity and stability [101], and their BGCs are one of the most abundant BGCs observed in *Streptomyces* along with those for ectoine [102]. Ectoine BGCs are highly conserved in *Streptomyces* and prevent osmotic stress [103]. In contrast, ectoine BGC was only observed in the genomes of *S. jiangxiensis* NBRC 100920^T^ and *S. oryzae* TH49^T^ with 23 and 100% gene content similarities to the reference BGCs in MIBiG database. No ectoine BGC was observed in the *Kitasatospora* genomes. These results indicate that *Streptacidiphilus* and *Kitasatospora* may have developed alternate mechanisms to carry out the functions otherwise performed by ectoines.

The other most abundant type of known BGC observed in *Streptacidiphilus* was that for macrotetrolide, a siderophore-type SM. Except *S. jiangxiensis* NBRC 100920^T^ and *S. bronchialis* DSM 106435^T^, each *Streptacidiphilus* species contained at least 1 (2 in the case of *S. albus* JL83^T^) macrotetrolide BGC and showed very low gene content similarities (25–33%) to the reference BGC (BGC0000244). Among the six representative *Streptomyces* and *Kitasatospora*, a macrotetrolide BGC was present in *Streptomyces albus* DSM 41398^T^ only, albeit as a part of hybrid butyrolactone-otherKS instead of a siderophore, as was found in the case of *Streptacidiphilus*.

Although macrotetrolides have been reported from *Streptomyces* [104], an extended search against an additional 129 complete *Streptomyces* genomes and 6 high-quality *Kitasatospora* genomes identified their presence in only seven *Streptomyces* genomes. No macrotetrolide BGC was detected in any *Kitasatospora* genome (unpublished data). The macrotetrolides are involved in a wide array of biological activities including antibacterial, antifungal, antitumor, antiprotozoan, antiparasitic, insecticidal and immunosuppressive activities [104].

Another most abundant known BGCs observed among *Streptacidiphilus* were those for echoside, which were present in 7 out of 11 genomes and exhibited limited gene content similarity (17–35%) with a reference MIBiG entry BGC0000340. No echoside BGC was observed in six representative species of other genera used in this study. When the genomes of 129 *Streptomyces* were scanned, only 17 such clusters were detected, and none of the additional *Kitasatospora* genomes contained echoside BGC (unpublished data). Echosides belong to *para*-terphenyl natural products and show inhibitory activities against DNA topoisomerase I and IIα, in addition to several other range of biological activities [105]. The majority of such compounds have been isolated from fungi, although a limited number of echosides have also been reported from some *Streptomyces*. Therefore, the presence of echoside BGCs in *Streptacidiphilus* may offer a rich source of potential novel compounds belonging to this family of natural products.

Despite the fact that the number of *Streptacidiphilus* genomes are limited at present, the overall distribution of known BGCs was different from *Streptomyces*. For example, BGCs such as those for albaflavenone and melanin which are otherwise abundant in *Streptomyces* [102] were not identified in any of the *Streptacidiphilus* species. On the other hand, only one melanin type BGC was found within the *Kitasatospora* (*Kitasatospora mediocidica* KCTC 9733^T^) genome.

Furthermore, there were at least seven known BGCs that were exclusively identified in *Streptacidiphilus* genomes. Two (lankacidin and cacibiocin) out of these seven known BGCs were detected in *S. pinicola* KCTC 49008^T^ and exhibited limited similarity with their corresponding reference BGCs in MIBiG database (Table 3). Overall, these results indicate that in addition to the core (present in all the species of three taxa) SM producing BGCs such as terpene and siderophore, there are some noticeable differences in the overall distribution of these BGCs within the family *Streptomycetaceae*.

### 3.6. Diversity of Carbohydrate-Active Enzymes

CAZymes play an essential role in the degradation as well as the biosynthesis of complex carbohydrates [38]. A wide range of organisms, including several species of *Streptomyces*, are known to produce these CAZymes [14] that can have environmental and industrial significance [106]. Recently, studies on the biodegradation potential of *Kitasatospora* have started to gain attention [16,17]. At present, there is only one *Streptacidiphilus* strain (*S. bronchialis* DSM 106435^T^) for which CAZyme annotations are available at CAZy database [38]. Therefore, in order to explore the diversity and distribution of CAZymes within *Streptacidiphilus*, the amino acid sequences of all strains belonging to any potential CAZy family were annotated by using the dbCAN2 meta server. On average, each strain of *Streptacidiphilus* contained 354 (±49.83) CAZy genes with one or more CAZy domains. A maximum number of 440 CAZy genes that encode various CAZymes were observed in *S. jeojiense* NRRL B-24555^T^ genome, whereas only 268 CAZyme encoding genes were present in *S. bronchialis* DSM 106435^T^. However, in terms of percentage, *S. carbonis* NBRC 100919^T^ was highest, with 5.98% of its genes containing one or more CAZy domains. Other species which contained ≥5% of CAZy genes included *S. jeojiense* NRRL B-24555^T^ (5.66%), *S. pinicola* KCTC 49008^T^ (5.24%), and *S. oryzae* TH49^T^ (5%). These numbers are impressive and highest among all the three types of taxonomic groups (Table 4) including a well-known cellulolytic strain *Streptomyces* sp. SirexAA-E [107]. A large number of these CAZymes consisted of glycoside hydrolase (GH) domains that represented a diverse set of CAZy families. This indicates that the genus *Streptacidiphilus*, akin to other representatives of the family *Streptomycetaceae* such as *Streptomyces*, has a potential to degrade biomass containing cellulose, chitin, etc. in acidic environments.

Specifically, the set of enzymes required for the degradation of at least three different types of complex carbohydrates, viz. cellulose, hemi-cellulose and chitin, were explored in more detail.

#### 3.6.1. Cellulose Degrading CAZymes or Cellulases

Cellulases are distributed in at least 11 different CAZy families, including GH5, GH6, GH7, GH8, GH9, GH12, GH44, GH45, GH48, GH61 and GH74 [108]. All 11 *Streptacidiphilus* strains contained at least one potential endoglucanase from GH5 and GH6 families. Specifically, GH5 enzymes were more abundant as compared to GH6 family, and could be as many as 11 in *S. carbonis* NBRC 100919^T^ or 9 in *S. pinicola* KCTC 49008^T^. These numbers are much higher in comparison to GH6 enzymes found in *Streptomyces* and *Kitasatospora* (Table 5). However, the distribution of endoglucanases from other GH families (e.g., GH8, GH9, GH12 and GH44) differed among strains and ranged between 0 and 2. Among all the species *S. bronchialis* DSM 106435^T^ possessed one additional endoglucanase from GH44 family. All *Kitasatospora* strains possessed one GH48 family, cellobiohydrolase, whereas some species of *Streptacidiphilus* and *Str. albus* DSM 41398^T^ lacked this family enzyme. Additionally, at least one endoglucanase or cellobiohydrolase from the GH6 (having both endoglucanase as well as cellobiohydrolase activity) family was present in all three taxonomic groups. None of these strains possessed endoglucanase- or cellobiohydrolase-encoding genes from GH45 and GH7 families. A large number (10–17) of β-glucosidases from GH1 and GH3 families were found in all *Streptacidiphilus* strains, and the number of β-glucosidases from family GH3 were higher as compared to GH1 (Table 5). For *Streptomyces*, the number of β-glucosidases ranged between 8 (*Str. avermitilis* MA-4680^T^) and 14 (*Str. coelicolor* A3(2)). *Kitasatospora*, on the other hand, consisted of a limited number of these enzymes with the exception of *K. azatica* KCTC 9699^T^, in which 12 β-glucosidases from GH1 and GH3 families were observed.

#### 3.6.2. Hemi-Cellulose Degrading CAZymes

The distribution of main hemi-cellulose degrading enzymes [109] in 11 *Streptacidiphilus* strains is summarized in Table 5. Notably, no endo-β-1,4-xylanase enzymes from families GH10, GH11 and GH30 were found in *S. albus* JL83^T^, while at least 10 genes that encode GH16 (xyloglycosyltransferase activity) family enzymes were present in its genome. This number is equal to the number of GH16 enzymes observed in *K. mediocidica* KCTC 9733^T^. Overall, the number of GH16 in *Kitasatospora* was higher than *Streptacidiphilus* and *Streptomyces*. In contrast, several endo-β-1,4-xylanase encoding genes including other xylan-degrading enzymes from different GH families were observed for *S. bronchialis* DSM 106435^T^ and *S. carbonis* NBRC 100919^T^ (Table 5). *S. carbonis* NBRC 100919^T^ genome contained a maximum of 45 potential hemicellulase degrading enzymes (belonging to all xylan degrading GH families), which is highest among all the species used in this study. The only other species in which all the potential xylan-degrading enzymes were observed was *K. azatica* KCTC 9699^T^, with 35 such genes.

Both cellulases and hemicellulases may contain additional carbohydrate binding modules (CBMs) that may bind to the substrates [109]. Additionally, other CAZymes including redox enzymes such as lytic polysaccharide monooxygenases belonging to family AA10, xylan esterases (CE), and polysaccharide lyases (PL) may also be involved in the degradation of cellulose and hemicellulose [110]. The AA10 family enzymes were abundant in *Streptomyces*, especially in *Str. coelicolor* A3(2) and *Streptomyces* sp. SirexAA, both well known for their glycan degrading activities. However, it can be noted from Table 5 that among *Streptacidiphilus*, only *S. bronchialis* DSM 106435^T^, a host associated strain [30], possessed three AA10 family CAZy enzymes. In the case of *Streptomyces*, it has been reported that host-associated strains may have higher cellulolytic activity [14]. Similarly, only *K. setae* KM-6054^T^ possessed enzymes belonging to AA10 family among the genus.

#### 3.6.3. Chitin Degrading CAZymes

Based on amino acid sequence similarities, chitin-degrading enzymes are divided into three CAZy families (GH18, GH19 and GH20) [111]. All these enzymes were observed in all strains with the exception of *S. oryzae* TH49^T^, *Str. avermitilis* MA-4680^T^, and *K. azatica* KCTC 9699^T^, which seemed to lack chitinase from the GH19 family. Specifically, 132 GH18 family chitinases were distributed among the 11 *Streptacidiphilus* strains, and the number was highest in *S. jeojiense* NRRL B-24555^T^ (17) and *S. jiangxiensis* NBRC 100920^T^ (16). Within the genomes of known carbohydrate degraders *Str. coelicolor* A3(2) and *Streptomyces* sp. SirexAA, 12 and 10 GH18 family enzymes were present. In contrast, *K. setae* KM-6054^T^ contained a maximum number of 19 GH18 enzymes among all species. When the amino acid sequences of 132 putative GH18 chitinases from *Streptacidiphilus* were compared with 80 chitinases from *Streptomyces* and *Kitasatospora*, the DXE catalytic motif [112] was well conserved in all these sequences despite a high degree of sequence variation (Figure S5). Further analysis of these 212 GH18 family sequences revealed that several of these enzymes could be grouped into 8 clusters based on their sequence identity (Figure S6). One sequence from *Str. coelicolor* A3(2) did not cluster with any of the sequences in the network and contained GH18 EndoS-like domain. This GH18 family endo-β-*N*-acetylglucosaminidase S (EndoS) is secreted by *Streptococcus pyogenes* and has the potential to hydrolyze the chitobiose core of asparagine-linked glycan on immunoglobulin G (IgG) [113]. The sequences in the well-defined clusters (clusters with 3 or more nodes) contained one or more domains in addition to the GH18 family domain and each cluster exhibited over-representation of certain domains. For example, several sequences of cluster 1 contained a fibronectin type 3 (FN3), or a chitin-binding domain of chitinase C (ChiC_BD) domains in addition to CBM_4_9 (annotated as CBM16 family by dbCAN) superfamily modules. In contrast, cluster 2 exhibited predominance of domains such as GH18_PF-ChiA-like, ricin-type β-trefoil lectin (ricin_B_lectin), and cellulose binding (CBM_2) domains. PF-ChiA is a chitinase present in the hyperthermophilic archaeon *Pyrococcus furiosus* with a GH18 catalytic activity. Although the catalytic residues are conserved in PF-ChiA, they mainly differ from the related GH18 family chitinases in their catalytic mechanism [114]. In addition to clusters 1 and 2, some sequences of cluster 3 also contained the FN3 domain, but the sequences in cluster 3 contained two FN3 domains instead of only one, as observed in the first two clusters. The FN3 domains are generally present between the catalytic and CBM modules [115].

## 4. Conclusions

This study provides the first analysis of high-quality genome sequences for *Streptacidiphilus*, and also the first comprehensive comparative genome analysis of eleven *Streptacidiphilus* and representative *Kitasatospora* and *Streptomyces* species, which highlighted the potential of this genus in terms of biosynthetic as well as biodegradation capabilities. In summary, we observe that hopene represents the core BGC in the family *Streptomcetaceae* since it was identified in the genome of each strain used in this study. Some BGCs such as macrotetrolide were more abundant in *Streptacidiphilus* as compared to *Streptomyces* and *Kitasatospora*. In contrast, albaflavenone and melanin, which are highly abundant in *Streptomyces*, were absent in *Streptacidiphilus*. Based on the similarities with existing BGCs, there is a high probability that the potential SMs produced by these *Streptacidiphilus* strains may be novel and the expected bioactivities may cover a wide range, including antibacterial, antifungal, antimalarial and antitumor activities. Similarly, some *Streptacidiphilus* species such as *S. carbonis* NBRC 100919^T^, *S. jeojiense* NRRL B-24555^T^, *S. pinicola* KCTC 49008^T^, and *S. oryzae* TH49^T^ exhibited a higher number of genes that may have implications in biodegradation. Moreover, our pan-genome analysis suggests an open pan-genome for *Streptacidiphilus* and highlights that the unique genome is the least annotated as compared to the core and accessory genome. The biosynthetic gene clusters or enzymes (especially those representing the unique genome) highlighted in this study would provide a starting point to explore the genus for its full potential. With the availability of more high-quality genomes from this genus, new insights regarding the developmental processes or the biosynthesis of secondary metabolites in *Streptomycetaceae* might be revealed in more detail.

## Figures and Tables

**Table 1:** Features of 11 *Streptacidiphilus* genomes and their comparison with representatives from *Streptomyces* and *Kitasatospora.* Species: 1, *Streptacidiphilus albus* JL83^T^; 2, *Streptacidiphilus anmyonensis* NBRC 103185^T^; 3, *Streptacidiphilus bronchialis* DSM 106435^T^; 4, *Streptacidiphilus carbonis* NBRC 100919^T^; 5, *Streptacidiphilus jeojiense* NRRL B-24555^T^; 6, *Streptacidiphilus jiangxiensis* NBRC 100920^T^; 7, *Streptacidiphilus melanogenes* NBRC 103184^T^; 8, *Streptacidiphilus neutrinimicus* NBRC 100921^T^; 9, *Streptacidiphilus oryzae* TH49^T^; 10, *Streptacidiphilus pinicola* KCTC 49008^T^; 11, *Streptacidiphilus rugosus* AM-16^T^; 12, *Streptomyces albus* DSM 41398^T^; 13, *Streptomyces avermitilis* MA-4680^T^; 14, *Streptomyces coelicolor* A3(2); 15, *Kitasatospora azatica* KCTC 9699^T^; 16, *Kitasatospora mediocidica* KCTC 9733^T^; 17, *Kitasatospora setae* KM-6054^T^.

Species	BioProject Accession	Size (Mb)	No. of Contigs	GC Content (%)	CDS	Pseudogenes	tRNA (rRNA)	Accessory Genes	Unique Genes	No. of Biosynthetic Gene Clusters (BGCs)	Reference
1	PRJNA234786	9.91	1	71.8	8,166	366	84 (27)	3,458	2,066	35	[5]
2	PRJDB3297	9.38	153	72	7941	453	73 (3)	4678	759	30	[23]
3	PRJNA438532	7.09	1	72.70	5681	247	66 (24)	1915	1665	19	[49]
4	PRJDB3297	8.45	139	71.3	7062	454	72 (3)	3591	1162	32	[5]
5	PRJNA238534	9.15	144	71.5	7769	257	73 (19)	3990	1376	18	-
6	PRJDB3297	9.52	96	72	8285	76	69 (5)	4545	1247	30	[50]
7	PRJDB3297	8.77	107	71.9	7481	390	74 (3)	4547	554	31	[23]
8	PRJDB3297	8.41	184	71.9	7119	459	71 (3)	4193	567	38	[5]
9	PRJNA234788	7.81	6	73.4	6526	272	63 (23)	2447	1884	15	[51]
10	PRJNA475452	8.43	281	71.8	7440	311	68 (8)	4137	846	35	[4]
11	PRJNA234778	9	4	71.8	7750	356	74 (27)	4101	1171	22	[23]
12	PRJNA271625	8.38	1	72.60	7330	58	65 (18)	NA	NA	35	[52]
13	PRJNA189	9.11	2	70.68	7446	0	68 (18)	NA	NA	37	[53]
14	PRJNA242	9.05	1	72	8152	60	65 (18)	NA	NA	29	[54]
15	PRJNA234862	8.27	3	71.6	6844	292	74 (29)	NA	NA	30	[55]
16	PRJNA234781	8.68	7	71.9	7103	254	71 (33)	NA	NA	32	[56]
17	PRJDA19951	8.78	1	74.20	7182	0	74 (27)	NA	NA	38	[57]

**Table 2:** Distribution of biosynthetic gene clusters (BGCs) in the genomes of *Streptacidiphilus* and their comparison with representative *Kitasatospora* and *Streptomyces.* Cluster type with an overall aggregate of 10 or above are shown only. Species: 1, *S. albus* JL83^T^; 2, *S. anmyonensis* NBRC 103185^T^; 3, *S. bronchialis* DSM 106435^T^; 4, *S. carbonis* NBRC 100919^T^; 5, *S. jeojiense* NRRL B-24555^T^; 6, *S. jiangxiensis* NBRC 100920^T^; 7, *S. melanogenes* NBRC 103184^T^; 8, *S. neutrinimicus* NBRC 100921^T^; 9, *S. oryzae* TH49^T^; 10, *S. pinicola* KCTC 49008^T^; 11, *S. rugosus* AM-16^T^; 12, *Str. albus* DSM 41398^T^; 13, *Str. avermitilis* MA-4680^T^; 14, *Str. coelicolor* A3(2); 15, *K. azatica* KCTC 9699^T^; 16, *K. mediocidica* KCTC 9733^T^; 17, *K. setae* KM-6054^T^.

Cluster Type/Species	1	2	3	4	5	6	7	8	9	10	11	12	13	14	15	16	17	Total
Terpene	3	4	3	6	5	6	4	4	8	5	5	2	7	5	3	3	5	78
T1PKS	0	6	0	8	1	1	6	11	0	3	0	3	4	1	2	5	1	52
NRPS	4	2	1	4	3	5	1	2	0	4	2	2	4	3	0	3	2	42
Siderophore	2	2	1	2	2	2	3	2	1	2	2	2	4	3	2	2	2	36
Lantipeptide	7	1	3	2	2	1	0	2	1	1	1	2	0	3	3	1	4	34
Bacteriocin	0	1	1	2	1	0	1	1	2	3	1	1	1	2	3	0	3	23
Butyrolactone	2	1	1	0	0	1	1	1	1	2	2	1	0	0	2	1	6	22
T2PKS	0	2	0	0	0	1	1	1	1	0	0	0	1	2	2	0	1	12
T3PKS	0	1	0	1	1	1	1	1	0	2	0	0	1	1	1	0	1	12
Lassopeptide	0	1	0	0	0	1	1	1	0	1	0	1	3	0	0	0	2	11
T1PKS_NRPS	1	0	3	1	0	1	0	1	0	0	0	1	1	0	1	0	0	10
Thiopeptide	2	0	1	0	0	0	0	0	0	2	1	1	0	0	2	0	0	9
T1PKS_OtherKS	0	0	0	0	0	0	0	0	0	0	0	2	1	2	1	1	1	8
Butyrolactone_OtherKS	0	0	0	0	0	1	1	0	0	1	0	1	1	0	0	1	0	6
Other hybrids	9	6	4	1	1	5	6	6	0	2	1	8	5	3	4	8	6	75
Others	0	0	1	0	0	0	0	1	1	1	2	5	3	3	1	3	2	23
Total	35	30	19	32	18	30	31	38	15	35	22	35	37	29	30	32	38	506

**Table 3:** List of known BGCs observed only in *Streptacidiphilus* genomes.

Known BGC	Organism Name	Cluster No.	Cluster Type	Similarity (%)	MIBiG Reference
Cacibiocin	*S. pinicola* KCTC 49008^T^	10	Aminocoumarin	64	BGC0001154
Cyanopeptin	*S. albus* JL83^T^	30	NRPS	50	BGC0000331
Eicoseicosapentaenoic acid	*S. bronchialis* DSM 106435^T^	18	Otherks	10	BGC0000865
Erythrochelin	*S. rugosus* AM-16^T^	18	NRPS	28	BGC0000349
Frenolicin	*S. jiangxiensis* NBRC 100920^T^	9	T2PKS	50	BGC0000225
Lankacidin	*S. pinicola* KCTC 49008^T^	25	Other	26	BGC0001100
Micromonolactam	*S. neutrinimicus* NBRC 100921^T^	2	T1PKS	100	BGC0000095

**Table 4:** Distribution of carbohydrate-active enzymes (CAZymes) and their various families in *Streptacidiphilus* and their comparison with representative *Streptomyces* and *Kitasatospora* species. For comparison, the CAZy profile of a well-known cellulose degrading *Streptomyces* sp. SirexAA strain is also shown. Values in parentheses (columns 3–8) represent different types of CAZy families. GH = glycoside hydrolase, GT = glycosyl transferase, CE = carbohydrate esterases, PL = polysaccharide lyases, CBM = carbohydrate-binding modules, AA = auxiliary activities.

Organism Name	Genes (%)	GH	GT	CE	PL	CBM	AA
*S. albus* JL83^T^	366 (4.48)	145 (46)	84 (14)	76 (7)	9 (6)	120 (16)	15 (5)
*S. anmyonensis* NBRC 103185^T^	339 (4.27)	134 (42)	79 (14)	71 (7)	4 (2)	108 (15)	24 (6)
*S. bronchialis* DSM 106435^T^	268 (4.72)	132 (49)	56 (13)	60 (8)	4 (3)	86 (20)	11 (4)
*S. carbonis* NBRC 100919^T^	422 (5.98)	235 (69)	77 (13)	77 (8)	9 (5)	152 (26)	15 (6)
*S. jeojiense* NRRL B-24555^T^	440 (5.66)	236 (60)	93 (12)	74 (8)	17 (5)	129 (21)	22 (6)
*S. jiangxiensis* NBRC 100920^T^	370 (4.47)	171 (56)	88 (14)	70 (8)	1 (1)	100 (18)	29 (6)
*S. melanogenes* NBRC 103184^T^	329 (4.40)	133 (43)	80 (14)	64 (8)	4 (3)	96 (16)	27 (6)
*S. neutrinimicus* NBRC 100921^T^	313 (4.40)	130 (47)	75 (14)	62 (7)	5 (4)	83 (16)	25 (6)
*S. oryzae* TH49^T^	326 (5.00)	154 (53)	83 (15)	64 (8)	4 (4)	48 (16)	20 (5)
*S. pinicola* KCTC 49008^T^	390 (5.24)	190 (61)	87 (13)	59 (9)	9 (7)	131 (20)	24 (5)
*S. rugosus* AM-16^T^	334 (4.31)	147 (47)	81 (14)	66 (8)	4 (4)	99 (16)	25 (6)
*Str. albus* DSM 41398^T^	262 (3.57)	120 (48)	55 (11)	54 (8)	3 (3)	30 (12)	21 (7)
*Str. avermitilis* MA-4680^T^	347 (4.52)	169 (55)	75 (13)	60 (9)	12 (9)	75 (18)	25 (7)
*Str. coelicolor* A3(2)	383 (4.70)	187 (60)	68 (14)	71 (9)	15 (11)	98 (19)	23 (7)
*Streptomyces* sp. sirexAA	297 (4.67)	137 (50)	63 (13)	63 (9)	8 (5)	80 (21)	17 (5)
*K. azatica* KCTC 9699^T^	364 (5.34)	172 (56)	70 (15)	67 (8)	2 (2)	160 (21)	18 (5)
*K. mediocidica* KCTC 9733^T^	333 (4.69)	128 (48)	84 (14)	71 (9)	8 (2)	96 (18)	15 (4)
*K. setae* KM-6054^T^	308 (4.29)	122 (41)	78 (13)	62 (8)	3 (2)	100 (19)	21 (5)

**Table 5:** Distribution of key CAZymes involved in the degradation of complex carbohydrates among *Streptacidiphilus* and their comparison with representative *Streptomyces* and *Kitasatospora.*

Enzyme Class	CAZy Family	Main Activity	*S. albus* JL83^T^	*S. anmyonensis* NBRC 103185^T^	*S. carbonis* NBRC 100919^T^	*S. jeojiense* NRRL B-24555^T^	*S. jiangxiensis* NBRC 100920^T^	*S. melanogenes* NBRC 103184^T^	*S. neutrinimicus* NBRC 100921^T^	*S. oryzae* TH49^T^	*S. pinicola* KCTC 49008^T^	*S. rugosus* AM-16^T^	*S. bronchialis* DSM 106435^T^	*Str. albus* DSM 41398^T^	*Str. avermitilis* MA-4680^T^	*Str. coelicolor* A3(2)	*Streptomyces* sp. SirexAA	*K*. *azatica* KCTC 9699^T^	*K*. *mediocidica* KCTC 9733^T^	*K*. *setae* KM-6054^T^
Cellulose degrading CAZymes	GH1	β-Glucosidase, β-galactosidase	4	3	5	3	3	6	3	3	4	6	4	3	3	5	6	5	2	4
	GH3	β-Glucosidases, β-D-xylopyranosidase	8	7	8	9	14	10	8	7	11	9	7	6	5	9	7	7	2	4
	GH5	Endo-β-1,4-glucanase, β-mannosidase	2	5	5	11	5	7	5	6	1	9	5	1	3	5	3	3	6	2
	GH6	Cellobiohydrolase, endo-β-1,4-glucanase	3	1	4	1	4	1	1	1	1	1	2	2	4	3	1	3	2	3
	GH8	Endo-β-1,4-glucanase	1	2	0	0	1	2	2	2	0	2	2	0	0	0	0	0	1	1
	GH9	Endo-β-1,4-glucanase	2	0	2	1	1	1	1	1	0	1	0	0	0	2	1	0	1	2
	GH12	Endo-β-1,4-glucanase	0	0	1	2	2	1	0	0	0	1	1	0	3	2	1	1	0	0
	GH44	Endoglucanase	0	0	1	0	0	0	0	0	0	0	0	0	0	0	0	0	0	0
	GH48	Cellobiohydrolase	1	0	1	1	1	1	0	0	1	1	1	0	1	1	1	1	1	1
	GH74	Endoglucanase	1	3	1	4	4	2	2	2	3	3	4	0	4	3	1	2	1	1
Subtotal			22	21	28	32	35	31	22	22	21	33	26	12	23	30	21	22	16	18
Hemicellulose degrading CAZymes	GH2	β-Galactosidase, β-glucuronidase	1	1	2	4	4	1	1	0	6	2	2	4	6	7	4	1	1	0
	GH10	Endo-β-1,4-xylanase	0	0	3	3	1	1	0	2	3	2	1	0	2	2	1	3	2	3
	GH11	Endo-β-1,4-xylanase	0	0	2	1	0	0	0	0	1	0	0	0	0	2	1	1	0	1
	GH16	Xyloglycosyltransferase	10	5	3	8	9	5	8	4	3	6	7	4	3	5	6	9	10	7
	GH26	Endo-β-1,4-mannanase	0	2	0	3	3	5	3	3	0	3	0	1	1	1	1	1	0	0
	GH30	Endo-β-1,4-xylanase	0	3	3	3	2	2	3	2	1	2	0	2	3	1	1	2	1	1
	GH31	α-Glucosidases, α-xylosidase	0	0	1	4	4	1	0	0	5	1	0	2	2	2	4	4	0	0
	GH39	α-L-iduronidase, β-xylosidase	3	0	0	2	2	1	1	1	2	0	2	0	2	0	0	1	0	0
	GH42	β-Galactosidase	0	2	2	6	4	5	2	1	2	6	2	0	4	2	3	2	1	0
	GH43	α-l-arabinofuranosidase, β-xylosidase	1	1	3	4	5	1	0	0	4	2	3	2	6	6	6	6	1	2
	GH53	Endo-β-1,4-galactanase	1	0	0	7	4	3	2	4	3	8	1	0	0	0	0	5	4	0
Subtotal			16	14	19	45	38	25	20	17	30	32	18	15	29	28	27	35	20	14
Chitin and chitosan degrading CAZymes	GH18	Chitinase	14	12	6	12	17	16	12	10	7	12	14	5	8	12	10	14	12	19
	GH19	Chitinase	1	2	2	1	2	2	1	1	0	1	1	1	0	2	3	0	1	2
	GH20	Exo-β-*N*-acetylglucosaminidase	2	4	2	3	3	5	4	2	4	4	4	5	3	4	2	2	2	2
	GH46	Endo-β-1,4-chitosanase	1	0	1	1	1	0	0	0	1	0	0	2	2	2	3	1	0	3
Subtotal			18	18	11	17	23	23	17	13	12	17	19	13	13	20	18	17	15	26
Oxidative enzymes	AA10	Lytic polysaccharide monooxygenase (LPMO)	0	0	3	0	0	0	0	0	0	0	0	1	4	7	6	0	0	5

## Supplementary Materials

The following are available online at https://www.mdpi.com/2073-4425/11/10/1166/s1, Figure S1. Whole genome-based tree of the family *Streptomycetaceae* generated with TYGS. *Streptomyces*, *Kitasatospora* and *Streptacidiphilus* are indicated as red, green and blue respectively. The tree was rooted at the midpoint, and GBDP bootstrap support values > 50% are indicated as black circles. Figure S2. Phylogeny of the amino acid sequences involved in the biosynthesis of cell wall peptidoglycan. (A) Enzymes encoded by two different types of *MurE* genes. (B) Amino acid sequences encoded by *dapF* genes in each strain. The trees were constructed with 1000 replicates of bootstrap test and distance estimates were calculated based on the Jones-Taylor-Thornton (JTT) model. Figure S3. NRPS type BGCs with well-defined modular structure as detected by antiSMASH in 3 high-quality (≤10 contigs) *Streptacidiphilus* genomes. Figure S4. Comparison of the antiSMASH predicted modular structures. (A) Scabichelin producing NRPS enzymes from *S. jeojiense* NRRL B-24555^T^ and *Streptomyces scabiei* 87.22. (B) hybrid T1PKS-NRPS cluster 5 from *Streptacidiphilus albus* JL83^T^ and the antimycin producing BGC from *Streptomyces* sp. S4. The predicted structures and potential monomers are also shown. Figure S5. Multiple sequence alignment between GH18 family chitinases from the representatives of family *Streptomycetaceae* showing highly conserved DXE catalytic motif. (X = any amino acid). Figure S6. Sequence similarity network of GH18 enzymes found in *Streptacidiphilus*, *Kitasatospora* and *Streptomyces*. In addition to GH18 domain, several other types of domains were observed in these enzymes. Based on their sequence similarity, GH18 enzymes were grouped into multiple clusters. Each node represents a GH18 enzyme and edges represent the sequence identity between the two nodes. Two nodes were connected if they shared ≥ 40% sequence identity. List of over-represented domains is provided under each cluster. Table S1. Average nucleotide identity (ANI) scores (%) observed between 11 *Streptacidiphilus* genomes and selected representatives of *Streptomyces* and *Kitasatospora*. Table S2. Pan-genome of *Streptacidiphilus* mapped to KEGG pathways. (A) Core genes. (B) Accessory genes. (C) Unique genes. Table S3. Diversity of core structures predicted by antiSMASH in *Streptacidiphilus.* Structures from BGCs which consisted of 5 or more genes are shown only. Table S4. Predicted core lanthipeptides detected by antiSMASH in *Streptacidiphilus*. (A) Class I lanthipeptides. (B) Class II lanthipeptides.
