# Supplementary material for: Genome Mining of the Genus Streptacidiphilus for Biosynthetic and Biodegradation Potential
Source: Genes (Basel). 2020 Oct 3;11(10):1166. doi: 10.3390/genes11101166 (PMC7601586; doi:10.3390/genes11101166)
Supplement: Supplementary file 1 [file genes-11-01166-s001.zip › Table-S1-final.docx]

**Table S1. Average nucleotide identity (ANI) scores (%) observed between 11 *Streptacidiphilus* genomes and selected representatives of *Streptomyces* and *Kitasatospora*.**

| **Strain** | 1 | 2 | 3 | 4 | 5 | 6 | 7 | 8 | 9 | 10 | 11 | 12 | 13 | 14 | 15 | 16 |
| --- | --- | --- | --- | --- | --- | --- | --- | --- | --- | --- | --- | --- | --- | --- | --- | --- |
| 1. *S. albus* JL83^T^ | - |  |  |  |  |  |  |  |  |  |  |  |  |  |  |  |
| 2. *S. anmyonensis* NBRC 103185^T^ | 78.2 | - |  |  |  |  |  |  |  |  |  |  |  |  |  |  |
| 3. *S. bronchialis* DSM 106435^T^ | 77.7 | 76.9 | - |  |  |  |  |  |  |  |  |  |  |  |  |  |
| 4. *S. carbonis* NBRC 100919*^T^* | 80.7 | 78.2 | 77.6 | - |  |  |  |  |  |  |  |  |  |  |  |  |
| 5. *S. jeojiense* NRRL B-24555^T^ | 80.6 | 78.1 | 77.6 | 88.2 | - |  |  |  |  |  |  |  |  |  |  |  |
| 6. *S. jiangxiensis* NBRC 100920^T^ | 78.2 | 85.3 | 76.7 | 78.1 | 78.1 | - |  |  |  |  |  |  |  |  |  |  |
| 7. *S. melanogenes* NBRC 103184^T^ | 78.1 | 90.1 | 76.7 | 78.2 | 78.1 | 85.4 | - |  |  |  |  |  |  |  |  |  |
| 8. *S. neutrinimicus* NBRC 100921^T^ | 78.1 | 90.0 | 76.9 | 78.2 | 78.1 | 85.6 | 91.1 | - |  |  |  |  |  |  |  |  |
| 9. *S. oryzae* TH49^T^ | 77.0 | 76.4 | 77.3 | 77.1 | 76.9 | 76.3 | 76.5 | 76.7 | - |  |  |  |  |  |  |  |
| 10. *S. pinicola* KCTC 49008^T^ | 78.0 | 86.6 | 76.9 | 78.5 | 78.3 | 85.8 | 86.8 | 86.8 | 76.6 | - |  |  |  |  |  |  |
| 11. *S. rugosus* AM-16^T^ | 78.5 | 82.3 | 77.1 | 78.7 | 78.6 | 82.2 | 82.3 | 82.7 | 76.6 | 82.4 | - |  |  |  |  |  |
| 12. *Str. albus* DSM 41398^T^ | 75.4 | 75.2 | 76.2 | 75.3 | 75.6 | 75.2 | 75.1 | 75.2 | 75.4 | 75.1 | 75.5 | - |  |  |  |  |
| 13. *Str.* *avermitilis* MA-4680^T^ | 74.6 | 74.9 | 75.5 | 74.7 | 74.7 | 74.7 | 74.7 | 74.9 | 74.7 | 74.6 | 74.7 | 78.9 | - |  |  |  |
| 14. *Str. coelicolor* A3(2) | 75.3 | 75.3 | 76.1 | 75.1 | 75.4 | 75.3 | 75.3 | 75.2 | 75.5 | 75.3 | 75.4 | 79.3 | 81.6 | - |  |  |
| 15. *K. azatica* KCTC 9699^T^ | 77.5 | 76.9 | 78.7 | 77.6 | 77.5 | 77.0 | 76.9 | 77.0 | 76.7 | 77.1 | 77.3 | 75.8 | 74.9 | 75.5 | - |  |
| 16. *K. mediocidica* KCTC 9733^T^ | 77.7 | 76.8 | 78.6 | 77.4 | 77.4 | 76.5 | 76.6 | 76.8 | 76.4 | 76.9 | 77.1 | 75.4 | 75.1 | 75.4 | 82.4 | - |
| 17. *K. setae* KM-6054^T^ | 77.3 | 76.8 | 78.5 | 77.1 | 77.2 | 76.7 | 76.6 | 76.9 | 76.9 | 76.8 | 77.1 | 75.8 | 75.1 | 75.7 | 79.9 | 79.5 |
