# Supplementary material for: Genome Mining of the Genus Streptacidiphilus for Biosynthetic and Biodegradation Potential
Source: Genes (Basel). 2020 Oct 3;11(10):1166. doi: 10.3390/genes11101166 (PMC7601586; doi:10.3390/genes11101166)
Supplement: Supplementary file 1 [file genes-11-01166-s001.zip › Table-S2-final.docx]

**Table S2. Pan-genome of *Streptacidiphilus* mapped to KEGG pathways.**

**(A) Core genes (≥5 counts)**

| **KEGG Pathway** | **Count** |
| --- | --- |
| 03010 Ribosome | 52 |
| 00230 Purine metabolism | 44 |
| 00190 Oxidative phosphorylation | 36 |
| 02020 Two-component system | 32 |
| 00860 Porphyrin and chlorophyll metabolism | 28 |
| 00010 Glycolysis / Gluconeogenesis | 26 |
| 00620 Pyruvate metabolism | 26 |
| 02010 ABC transporters | 25 |
| 00720 Carbon fixation pathways in prokaryotes | 24 |
| 00250 Alanine, aspartate and glutamate metabolism | 23 |
| 00520 Amino sugar and nucleotide sugar metabolism | 21 |
| 00240 Pyrimidine metabolism | 21 |
| 00970 Aminoacyl-tRNA biosynthesis | 21 |
| 00020 Citrate cycle (TCA cycle) | 20 |
| 00650 Butanoate metabolism | 20 |
| 00260 Glycine, serine and threonine metabolism | 20 |
| 00280 Valine, leucine and isoleucine degradation | 20 |
| 00630 Glyoxylate and dicarboxylate metabolism | 19 |
| 02024 Quorum sensing | 19 |
| 00680 Methane metabolism | 17 |
| 00500 Starch and sucrose metabolism | 16 |
| 00640 Propanoate metabolism | 15 |
| 00360 Phenylalanine metabolism | 15 |
| 00400 Phenylalanine, tyrosine and tryptophan biosynthesis | 15 |
| 00220 Arginine biosynthesis | 14 |
| 00340 Histidine metabolism | 14 |
| 00770 Pantothenate and CoA biosynthesis | 14 |
| 03440 Homologous recombination | 14 |
| 00550 Peptidoglycan biosynthesis | 13 |
| 00760 Nicotinate and nicotinamide metabolism | 13 |
| 00270 Cysteine and methionine metabolism | 12 |
| 00290 Valine, leucine and isoleucine biosynthesis | 12 |
| 00310 Lysine degradation | 12 |
| 00300 Lysine biosynthesis | 11 |
| 00790 Folate biosynthesis | 11 |
| 03018 RNA degradation | 11 |
| 03030 DNA replication | 11 |
| 03410 Base excision repair | 11 |
| 00030 Pentose phosphate pathway | 10 |
| 00051 Fructose and mannose metabolism | 10 |
| 00710 Carbon fixation in photosynthetic organisms | 10 |
| 00564 Glycerophospholipid metabolism | 10 |
| 00330 Arginine and proline metabolism | 10 |
| 00730 Thiamine metabolism | 10 |
| 00900 Terpenoid backbone biosynthesis | 10 |
| 04122 Sulfur relay system | 10 |
| 00071 Fatty acid degradation | 9 |
| 00380 Tryptophan metabolism | 9 |
| 00410 beta-Alanine metabolism | 9 |
| 00670 One carbon pool by folate | 9 |
| 00130 Ubiquinone and other terpenoid-quinone biosynthesis | 9 |
| 03060 Protein export | 9 |
| 03430 Mismatch repair | 9 |
| 00920 Sulfur metabolism | 8 |
| 00780 Biotin metabolism | 8 |
| 03420 Nucleotide excision repair | 8 |
| 03070 Bacterial secretion system | 8 |
| 00660 C5-Branched dibasic acid metabolism | 7 |
| 00061 Fatty acid biosynthesis | 7 |
| 00350 Tyrosine metabolism | 7 |
| 02025 Biofilm formation - Pseudomonas aeruginosa | 7 |
| 04922 Glucagon signaling pathway | 7 |
| 01501 beta-Lactam resistance | 7 |
| 00480 Glutathione metabolism | 6 |
| 00983 Drug metabolism - other enzymes | 6 |
| 04066 HIF-1 signaling pathway | 6 |
| 04112 Cell cycle - Caulobacter | 6 |
| 00040 Pentose and glucuronate interconversions | 5 |
| 00052 Galactose metabolism | 5 |
| 00562 Inositol phosphate metabolism | 5 |
| 00195 Photosynthesis | 5 |
| 00561 Glycerolipid metabolism | 5 |
| 00450 Selenocompound metabolism | 5 |
| 00740 Riboflavin metabolism | 5 |
| 00362 Benzoate degradation | 5 |
| 04146 Peroxisome | 5 |
| 05111 Biofilm formation - Vibrio cholerae | 5 |
| 02026 Biofilm formation - Escherichia coli | 5 |
| 03320 PPAR signaling pathway | 5 |
| 04212 Longevity regulating pathway - worm | 5 |
| 05230 Central carbon metabolism in cancer | 5 |
| 01502 Vancomycin resistance | 5 |
| Others | 190 |
| Total | 1,254 |

**(B) Accessary genes (≥5 counts)**

| **KEGG Pathway** | **Count** |
| --- | --- |
| 02010 ABC transporters | 119 |
| 02020 Two-component system | 74 |
| 00520 Amino sugar and nucleotide sugar metabolism | 42 |
| 02024 Quorum sensing | 35 |
| 00230 Purine metabolism | 30 |
| 00330 Arginine and proline metabolism | 29 |
| 00620 Pyruvate metabolism | 27 |
| 00270 Cysteine and methionine metabolism | 26 |
| 00260 Glycine, serine and threonine metabolism | 25 |
| 00010 Glycolysis / Gluconeogenesis | 24 |
| 00630 Glyoxylate and dicarboxylate metabolism | 24 |
| 00500 Starch and sucrose metabolism | 22 |
| 00640 Propanoate metabolism | 21 |
| 00030 Pentose phosphate pathway | 20 |
| 00240 Pyrimidine metabolism | 20 |
| 00860 Porphyrin and chlorophyll metabolism | 20 |
| 00650 Butanoate metabolism | 19 |
| 00250 Alanine, aspartate and glutamate metabolism | 19 |
| 00220 Arginine biosynthesis | 19 |
| 00362 Benzoate degradation | 19 |
| 00040 Pentose and glucuronate interconversions | 18 |
| 00051 Fructose and mannose metabolism | 18 |
| 00680 Methane metabolism | 18 |
| 00920 Sulfur metabolism | 18 |
| 00280 Valine, leucine and isoleucine degradation | 18 |
| 00360 Phenylalanine metabolism | 18 |
| 00071 Fatty acid degradation | 17 |
| 00130 Ubiquinone and other terpenoid-quinone biosynthesis | 17 |
| 00350 Tyrosine metabolism | 16 |
| 00970 Aminoacyl-tRNA biosynthesis | 16 |
| 00052 Galactose metabolism | 14 |
| 00720 Carbon fixation pathways in prokaryotes | 14 |
| 00561 Glycerolipid metabolism | 14 |
| 00380 Tryptophan metabolism | 14 |
| 00910 Nitrogen metabolism | 13 |
| 00190 Oxidative phosphorylation | 12 |
| 00061 Fatty acid biosynthesis | 12 |
| 00400 Phenylalanine, tyrosine and tryptophan biosynthesis | 12 |
| 00550 Peptidoglycan biosynthesis | 11 |
| 00760 Nicotinate and nicotinamide metabolism | 11 |
| 03030 DNA replication | 11 |
| 00020 Citrate cycle (TCA cycle) | 10 |
| 00562 Inositol phosphate metabolism | 10 |
| 00564 Glycerophospholipid metabolism | 10 |
| 00300 Lysine biosynthesis | 10 |
| 00410 beta-Alanine metabolism | 10 |
| 00480 Glutathione metabolism | 10 |
| 00627 Aminobenzoate degradation | 10 |
| 00983 Drug metabolism - other enzymes | 10 |
| 04142 Lysosome | 10 |
| 04146 Peroxisome | 10 |
| 00310 Lysine degradation | 9 |
| 00340 Histidine metabolism | 9 |
| 00460 Cyanoamino acid metabolism | 9 |
| 00521 Streptomycin biosynthesis | 9 |
| 00625 Chloroalkane and chloroalkene degradation | 9 |
| 03430 Mismatch repair | 9 |
| 03440 Homologous recombination | 9 |
| 02025 Biofilm formation - Pseudomonas aeruginosa | 9 |
| 02026 Biofilm formation - Escherichia coli | 9 |
| 00053 Ascorbate and aldarate metabolism | 8 |
| 00450 Selenocompound metabolism | 8 |
| 00511 Other glycan degradation | 8 |
| 00900 Terpenoid backbone biosynthesis | 8 |
| 00984 Steroid degradation | 8 |
| 03410 Base excision repair | 8 |
| 01501 beta-Lactam resistance | 8 |
| 00600 Sphingolipid metabolism | 7 |
| 00571 Lipoarabinomannan (LAM) biosynthesis | 7 |
| 00780 Biotin metabolism | 7 |
| 00670 One carbon pool by folate | 7 |
| 00830 Retinol metabolism | 7 |
| 00361 Chlorocyclohexane and chlorobenzene degradation | 7 |
| 03018 RNA degradation | 7 |
| 04112 Cell cycle - Caulobacter | 7 |
| 01503 Cationic antimicrobial peptide (CAMP) resistance | 7 |
| 00710 Carbon fixation in photosynthetic organisms | 6 |
| 01040 Biosynthesis of unsaturated fatty acids | 6 |
| 00430 Taurine and hypotaurine metabolism | 6 |
| 00740 Riboflavin metabolism | 6 |
| 00750 Vitamin B6 metabolism | 6 |
| 00770 Pantothenate and CoA biosynthesis | 6 |
| 00790 Folate biosynthesis | 6 |
| 00405 Phenazine biosynthesis | 6 |
| 00999 Biosynthesis of secondary metabolites - unclassified | 6 |
| 05111 Biofilm formation - Vibrio cholerae | 6 |
| 03320 PPAR signaling pathway | 6 |
| 00290 Valine, leucine and isoleucine biosynthesis | 5 |
| 00730 Thiamine metabolism | 5 |
| 00903 Limonene and pinene degradation | 5 |
| 00523 Polyketide sugar unit biosynthesis | 5 |
| 00940 Phenylpropanoid biosynthesis | 5 |
| 00791 Atrazine degradation | 5 |
| 00626 Naphthalene degradation | 5 |
| 00980 Metabolism of xenobiotics by cytochrome P450 | 5 |
| 03060 Protein export | 5 |
| Others | 350 |
| Total | 1,707 |

**(C) Unique genes (≥5 counts)**

| **KEGG Pathway** | **Count** |
| --- | --- |
| 02010 ABC transporters | 68 |
| 02020 Two-component system | 40 |
| 00620 Pyruvate metabolism | 32 |
| 02024 Quorum sensing | 28 |
| 00010 Glycolysis / Gluconeogenesis | 26 |
| 00520 Amino sugar and nucleotide sugar metabolism | 24 |
| 00640 Propanoate metabolism | 24 |
| 00650 Butanoate metabolism | 24 |
| 00230 Purine metabolism | 24 |
| 00260 Glycine, serine and threonine metabolism | 22 |
| 00360 Phenylalanine metabolism | 21 |
| 00330 Arginine and proline metabolism | 20 |
| 00680 Methane metabolism | 19 |
| 00630 Glyoxylate and dicarboxylate metabolism | 18 |
| 00720 Carbon fixation pathways in prokaryotes | 18 |
| 00250 Alanine, aspartate and glutamate metabolism | 18 |
| 00500 Starch and sucrose metabolism | 17 |
| 00071 Fatty acid degradation | 17 |
| 00280 Valine, leucine and isoleucine degradation | 17 |
| 00270 Cysteine and methionine metabolism | 15 |
| 00350 Tyrosine metabolism | 15 |
| 00020 Citrate cycle (TCA cycle) | 14 |
| 00920 Sulfur metabolism | 14 |
| 00410 beta-Alanine metabolism | 14 |
| 00310 Lysine degradation | 13 |
| 00380 Tryptophan metabolism | 13 |
| 00362 Benzoate degradation | 13 |
| 00030 Pentose phosphate pathway | 12 |
| 00040 Pentose and glucuronate interconversions | 12 |
| 00130 Ubiquinone and other terpenoid-quinone biosynthesis | 12 |
| 00970 Aminoacyl-tRNA biosynthesis | 12 |
| 00051 Fructose and mannose metabolism | 11 |
| 00061 Fatty acid biosynthesis | 11 |
| 00220 Arginine biosynthesis | 11 |
| 00984 Steroid degradation | 11 |
| 00770 Pantothenate and CoA biosynthesis | 10 |
| 00627 Aminobenzoate degradation | 10 |
| 00052 Galactose metabolism | 9 |
| 00910 Nitrogen metabolism | 9 |
| 00561 Glycerolipid metabolism | 9 |
| 00240 Pyrimidine metabolism | 9 |
| 00340 Histidine metabolism | 9 |
| 00400 Phenylalanine, tyrosine and tryptophan biosynthesis | 9 |
| 00550 Peptidoglycan biosynthesis | 9 |
| 00790 Folate biosynthesis | 9 |
| 00860 Porphyrin and chlorophyll metabolism | 9 |
| 01053 Biosynthesis of siderophore group nonribosomal peptides | 9 |
| 00564 Glycerophospholipid metabolism | 8 |
| 00300 Lysine biosynthesis | 8 |
| 00450 Selenocompound metabolism | 8 |
| 00511 Other glycan degradation | 8 |
| 04142 Lysosome | 8 |
| 00053 Ascorbate and aldarate metabolism | 7 |
| 00562 Inositol phosphate metabolism | 7 |
| 00760 Nicotinate and nicotinamide metabolism | 7 |
| 00625 Chloroalkane and chloroalkene degradation | 7 |
| 00710 Carbon fixation in photosynthetic organisms | 6 |
| 00290 Valine, leucine and isoleucine biosynthesis | 6 |
| 00480 Glutathione metabolism | 6 |
| 00780 Biotin metabolism | 6 |
| 01054 Nonribosomal peptide structures | 6 |
| 00521 Streptomycin biosynthesis | 6 |
| 04122 Sulfur relay system | 6 |
| 04146 Peroxisome | 6 |
| 00600 Sphingolipid metabolism | 5 |
| 01040 Biosynthesis of unsaturated fatty acids | 5 |
| 00430 Taurine and hypotaurine metabolism | 5 |
| 00531 Glycosaminoglycan degradation | 5 |
| 00900 Terpenoid backbone biosynthesis | 5 |
| 00626 Naphthalene degradation | 5 |
| 00982 Drug metabolism - cytochrome P450 | 5 |
| 03030 DNA replication | 5 |
| 03430 Mismatch repair | 5 |
| 04112 Cell cycle - Caulobacter | 5 |
| 02025 Biofilm formation - Pseudomonas aeruginosa | 5 |
| 02030 Bacterial chemotaxis | 5 |
| 01501 beta-Lactam resistance | 5 |
| Others | 298 |
| Total | 1,269 |
