# Supplementary material for: Genome Mining of the Genus Streptacidiphilus for Biosynthetic and Biodegradation Potential
Source: Genes (Basel). 2020 Oct 3;11(10):1166. doi: 10.3390/genes11101166 (PMC7601586; doi:10.3390/genes11101166)
Supplement: Supplementary file 1 [file genes-11-01166-s001.zip › Table-S3-final.docx]

**Table S3. Diversity of core structures predicted by antiSMASH in *Streptacidiphilus*.**

Structures from BGCs which consisted of 5 or more genes are shown only.

| Organism | Cluster | Predicted Monomers^1^ | Predicted Core Structure |
| --- | --- | --- | --- |
| (A) T1PKS | | | |
| *S. anmyonensis* NBRC 103185^T^ | 4 | (mal) + (Ala) | 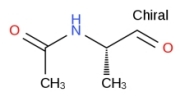 |
|  | 7 | (pk) + (ccmmal) | 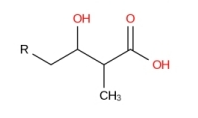 |
|  | 10 | (ccmal) + (ccmal-ccmal) + (pk) + (Ala) + (mal) + (ohmal-ccmal) + (ccmal) |  |
|  | 17 | (mal) + (pk-ccmal-ccmal) + (ccmal-ccmal) + (ohmmal-ohmmal) + (ccmal-redmal) + (Asn) + (mal) |  |
| *S. carbonis* NBRC 100919^T^ | 15 | (mmal-redmal-redmal) + (ohmal) + (mal) + (ccmmal-ccmmal-ohmal) + (redmal) | 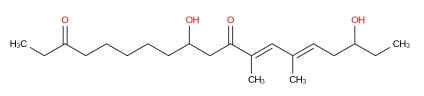 |
| *S. jiangxiensis* NBRC 100920^T^ | 22 | (mmal) + (mmal-redmal) + (mmal) + (ccmal) + (ccmal) | 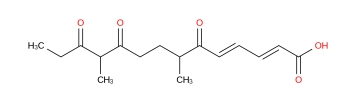 |
| *S. melanogenes* NBRC 103184^T^ | 3 | (ccmal) + (ccmal) + (ohmmal-ohmmal) + (redmal) | 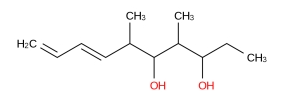 |
|  | 4 | (ohmal-ccmal) + (pk) | 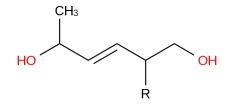 |
|  | 6 | (ccmal-ccmal) + (pk) + (Ala) + (ccmal) + (pk) + (ccmal) | 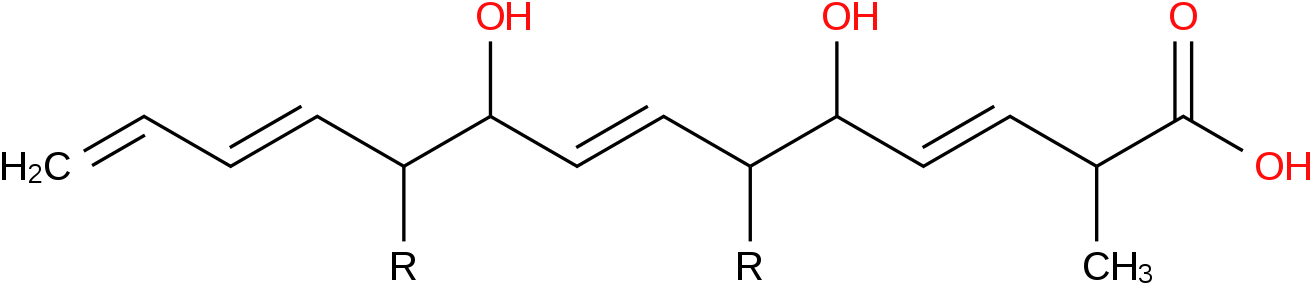 |
|  | 9 | (redmmal) + (Arg) | 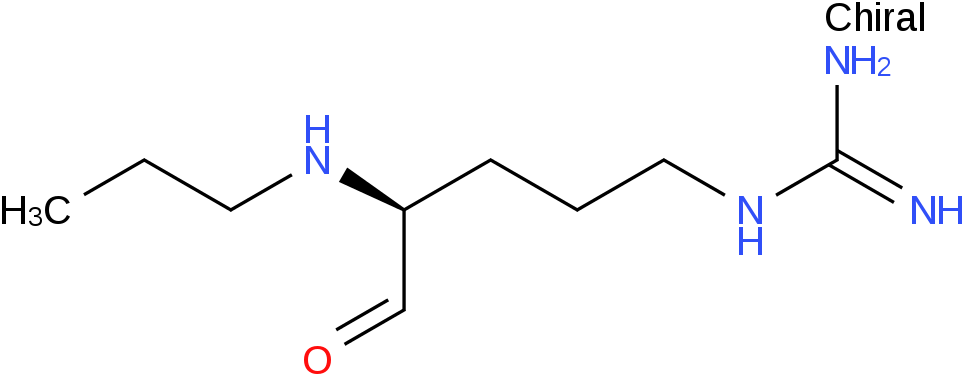 |
| *S. neutrinimicus* NBRC 100921^T^ | 9 | (ohmal) + (ohmal) + (pk) | 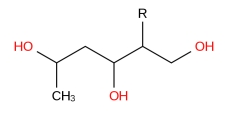 |
|  | 10 | (ccmal-mal) + (Asn) | 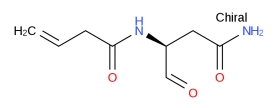 |
|  | 14 | (ccmal) + (pk) + (Ala) + (mal) | 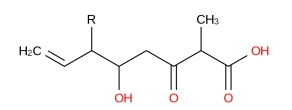 |
| *S. pinicola* KCTC 49008^T^ | 20 | (mal-ccmal) + (mal) + (mal) + (pk) | 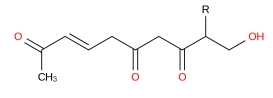 |
|  | 23 | (mal) + (Ala) | 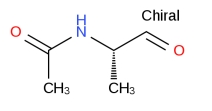 |
| (B) NRPS | | | |
| *S. albus* JL83^T^ | 9 | (Arg) + (Thr) + (Leu\|Val) | 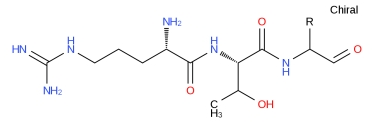 |
|  | 26 | (Leu-Ala-Val-Leu) + (orn-Thr-Lys-Leu\|Val-Leu) | 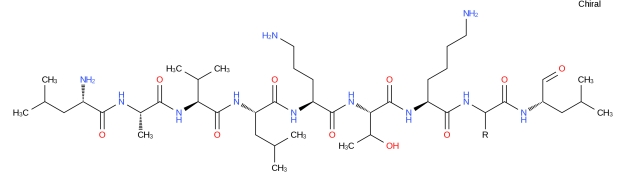 |
|  | 28 | (Ala) + (Ala) | 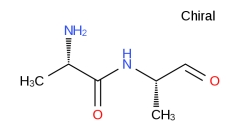 |
|  | 30 | (Leu) + (Leu-Leu) + (Ala-Ser) + (Ala-Ala) + (Ala) | 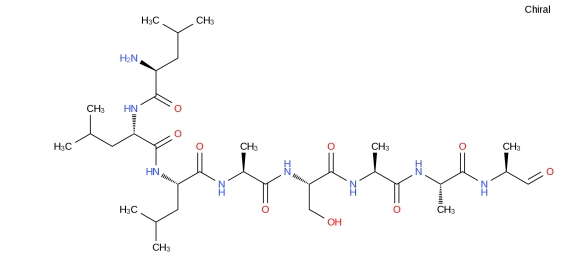 |
| *S. anmyonensis* NBRC 103185^T^ | 15 | (hpg\|hpg2cl) + (Ala-Pro-Ala) | 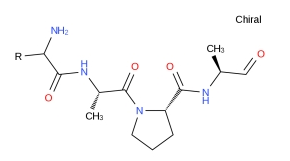 |
|  | 16 | (Ala) + (Ala) + (Ala) + (Ala) | 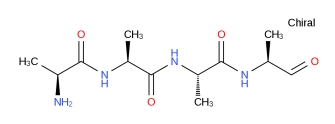 |
| *S. bronchialis* DSM 106435^T^ | 17 | (Ala) + (orn-Thr-Ala-Pro-Phe-Bht-Leu\|Val) + (Ala) | 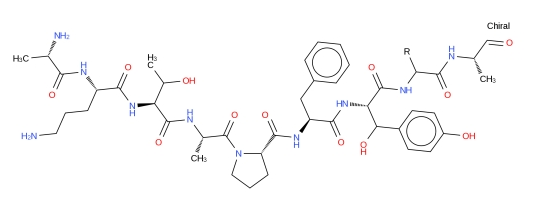 |
| *S. carbonis* NBRC 100919^T^ | 7 | (Leu-Ala) | 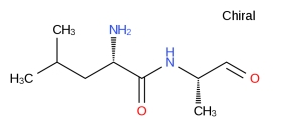 |
| *S. jeojiense* NRRL B-24555^T^ | 6 | (Val) + (Val) + (Leu-orn-Phe) + (Ala) + (dhb\|sal) | 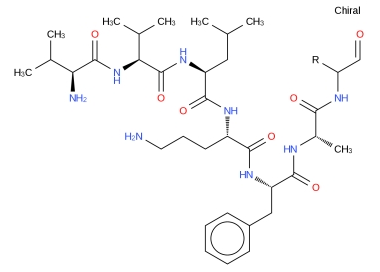 |
|  | 8 | (Trp-Gln-horn-Ser-Ser-orn) | 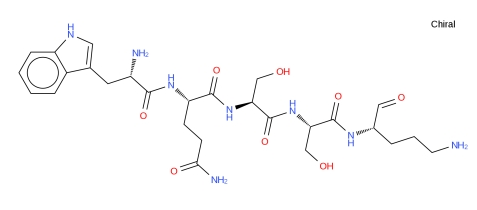 |
| *S. jiangxiensis* NBRC 100920^T^ | 2 | (Val) + (Thr) | 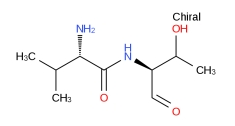 |
|  | 5 | (Gly-Ala) + (Ala) + (Asn) + (Ala) | 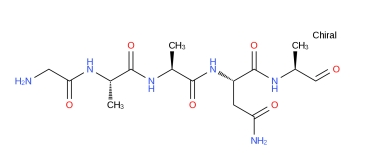 |
|  | 21 | (Ala-Gly-Ser-Ala-Ala-Ala) + (Val-Ala-Thr-Val) | 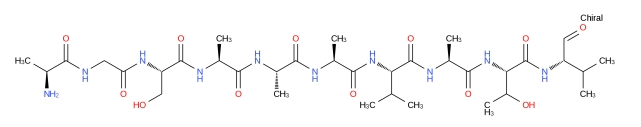 |
|  | 27 | (Gly) + (Gly) + (Ala) + (Ser) | 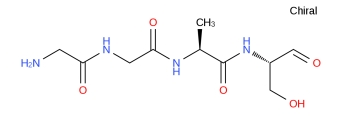 |
| *S. neutrinimicus* NBRC 100921^T^ | 25 | (Ala) + (Leu-Ala-Leu\|Val-Ala-Leu) + (Lys-aThr\|Thr-Lys-Leu\|Val-Leu) | 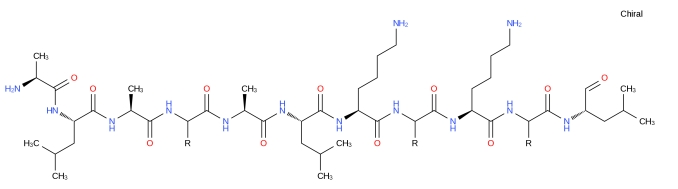 |
| *S. pinicola* KCTC 49008^T^ | 4 | (Ala) + (Leu-orn-Leu) | 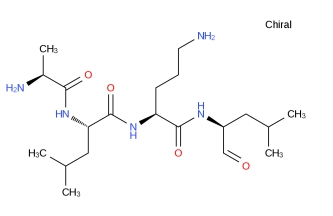 |
|  | 6 | (Val) + (Val) | 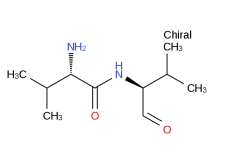 |
|  | 29 | (Ala) + (pip-Ala-Ala-Ala) + (Cys-Ala) | 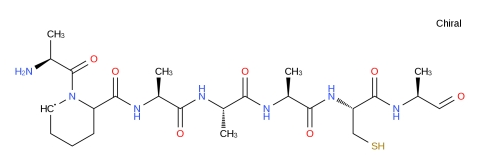 |
| *S. rugosus* AM-16^T^ | 18 | (Val) + (Lys-Leu-Ser) + (Leu) + (Ala) | 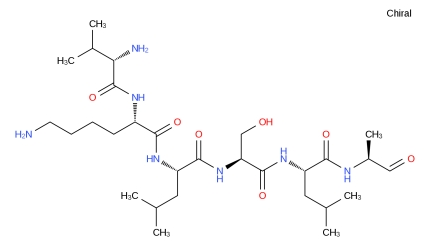 |
| (C) T1PKS-NRPS hybrid | | | |
| *S. albus* JL83^T^ | 5 | (Ala) + (Pro-Ala-Ala-Tyr) + (b-Ala) + (mal) + (dhab\|dht\|Thr-Ala) + (Ala) + (mmal) + (Ala) | 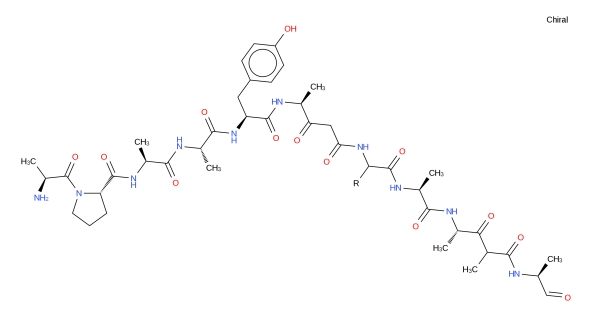 |
| *S. bronchialis* DSM 106435^T^ | 14 | (ccmal) + (redmmal) + (pk) + (ccmal) + (Cys) + (Cys) + (Cys) | 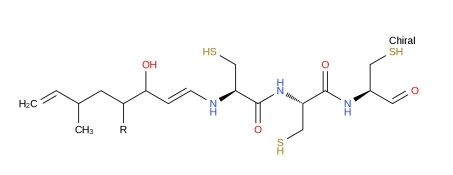 |
|  | 15 | (Ala-Thr-orn) + (ccmal-ccmal) + (ccmal) + (pk) + (pk) + (redmmal) + (mal) | 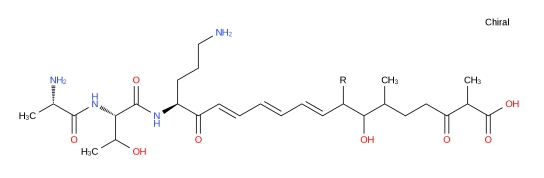 |
| *S. carbonis* NBRC 100919^T^ | 14 | (redmal) + (Ile) + (Ala-Val-pip-d-Trp) + (Phe-Ala) | 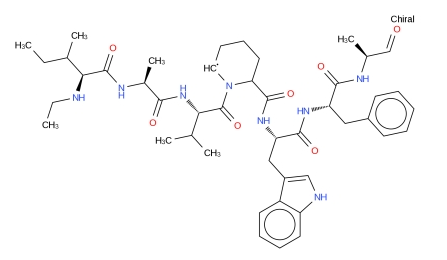 |
| *S. jiangxiensis* NBRC 100920^T^ | 26 | (pk-mmal) | 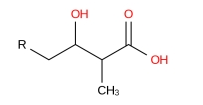 |
| *S. neutrinimicus* NBRC 100921^T^ | 23 | (Val) + (mal) + (Thr) | 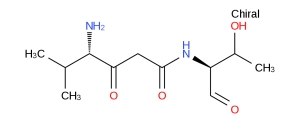 |

^1^ For the description of monomers, interested readers are referred to [107116] and Norine [108117] database (https://bioinfo.lifl.fr/norine/listAmino.js).
