# Supplementary material for: Genome Mining of the Genus Streptacidiphilus for Biosynthetic and Biodegradation Potential
Source: Genes (Basel). 2020 Oct 3;11(10):1166. doi: 10.3390/genes11101166 (PMC7601586; doi:10.3390/genes11101166)
Supplement: Supplementary file 1 [file genes-11-01166-s001.zip › Table-S4-final.docx]

**Table S4. Predicted core lanthipeptides detected by antiSMASH in *Streptacidiphilus*.**

**A. Class I lanthipeptides**

| **Strain** | **Cluster** | **Predicted core peptide** |
| --- | --- | --- |
| *S. albus* JL83^T^ | 10 | - MAPTAPSSLDRTTGADVDPLDLDLTVITEVGTAAIPG – CDDhbDhaDGCGDhaDhbCADhaACNDhaAV |
|  | 21 | - MAPSTATLEPAIRTGAASDDFDDLDVSVVEVLDPA - HLVDhaMDhbDDNCGDhaDhbCEKDhaDhbCIDhaAV |
|  | 23 | - MTAMLTEHPPTETEDPFALDVRVVVAAHPGGK – LMCDhaDhbGDGCGDhbDhbCADhbGADhaACNDhaDhaDhbEEPDha |
| *S.* *bronchialis* DSM 106435**^T^** | 8 | - MADTRVSGGATALAERPAADGAFADEFSLDVRVVVAAHPNGKLACS – DhbDhaDGCGDhaDhaCDhaGDhaACDhbDhaFDhbDDPV |
|  | 10 | - MAPQSSTLKAATDFSGEGMSDFDLSIETVASAPIVPG - LLNDDhbGDGCGDhaDhbCQDhaACDhaNDhaDhbCIDhaG |
|  | 12 | - MTVQMEPPVAPAQPTAEQQDLTGFDLDITIVEGGPAAD – QLIRLDhbDDGCNDhaDhbCADhbACVDhaCP |
| *S. carbonis* NBRC 100919^T^ | 22 | - MNAVLGEPMALDAADLFDLDVEFVAMSGDTDLSAL – DhaDhbPDhbDhaDGDhaACYNDhaMCCNDhaNWGC |
| *S. jeojiense* NRRL B-24555^T^ | 18 | - MNSTEDFDLDVSFADR – GdhbVIGELLNDhaDhbDhaDGCGDhaDhbGADhaACAGCVDhbD |
| *S. jiangxiensis* NBRC 100920^T^ | 15 | - MSTPTITPPSTKPDELASPAANPFELDIHFIEGTPESETV – LMCGDhbGDNCGDhaDhaCPDhaACADhbDha |

**B. Class II lanthipeptides**

| **Strain** | **Cluster** | **Predicted core peptide** |
| --- | --- | --- |
| *S. albus* JL83^T^ | 6 | - MHTVLEESQLTIAAWRDGTGTDSPAGPLFTGGAYAESEIAA - DhaDQVKDhbVQCGDhbVCDhaYDhaAGRDCC |
|  | 24 | - VTSESHDESFS –PCDhbDNFRDhbGVYQICPDNDhaLLAIDhaDhaYRVHADhaPV - MSNEALVTGWKNPAAREQPGPGHPAGDIRLPQESAAGRRARLLAG – LADhaLDhaCNDVNDhbVDhbIDhbDhbIDhaMDhaWG - MVHGSPAVVAARLDALAAELGA – DEIMLAPYELDhbGPARCRDhbLRLAAGVRRPRARLLGDhbADhaRDha - VAENIEPTVETPEDDAPEVEAHAEQVLGLQGISIDKETQEGLMVAPGS – CdhaDhaCAGDhaLCN - MVTGAEAVPLPWG – DdhaVPVQVVPEPKADhaVWPAVKDhbVALDhbDhaLDhaELYGDDRVPLPDhaLGCEQDhaDhb - VTTESGPVPQTPSCSTGDCGTGPSRWCGPRGGVGRAGRAGHSYLGM – ANLDhaDhaRCLDhaIRQRGDhaPPCPCP |
|  | 22 | - MNTGRPTPAIRQRSGTGA – AGEYGDhbAPDhaCP - LQPWMCRHGAYRSRDPEPTASVAG – KVGAVPCNRLPQYEAVALDGQRDhbDhbVPDha - MGAHGVDRCTRPAGVAERQLGVSPRTWRTCPSAAPQQMDAPSEQAFAGG – RPVDVDhaGDCAVVAKIDhaFAQVCDha - MTNPFENEDGTYLVLVNDENQHSLWPEQIELPAG – WdhaDhbAHGPAARDhbECLDFINVDhbWDhbDMRPRDhaLAALMDQAR - VAVVADAGSDLAGS – ARRAVGDhbAAPRRRAADRCGARRARRGPAPDPMGPRCPG - VRRGGGEEGRAPAG – DhbVDPALDhbADhbGCQDhbFLQL - VFRFRAPAPNLGDGGGG – PENDhaGRGIRFARRYPCPADhaAVPPEVRCHLKDha - VAPPTRGTGIGG – RIGCRGRNFRARRHRHRG - VDTAPGDRDFPTPIANGGRVHASSPGG – DhbNRPACVHVDhaEPAVEHPNDhbPDhaDhbPAGPRPADLRDhaRIA - MCVILPSQVLTCELLLPPEPMTSLMRFLASGNDVRSRLRSKSSPAA – LAWIRAC - LLALRPLTLRPYSRATGRTSADAVREAGESEGP – NWEDCPPMPRGIDEAR - LLADLCPPGVRTVRPGAGEKRLPFM – AGVLQRLRVFRECPV - MSGLITGCRAGRARRRGRRPGA – ERVRPCAAG - VLGTALLLRRWPTRRRGRHGG – AdhaWCGRHGAGPVHRDhaLMGPHQPDhaADhaRWLDhaRDhaWPRLRPV |
| *S. anmyonensis* NBRC 103185^T^ | 9 | - VREFDGGRAGA – GRAPLPHRRRRIDhaRGWAAWWDhaCDhaDhaRWDhaCW - MGGGRRKHGAVSGM - DhaVIDhbGCC |
| *S. melanogenes* NBRC 103184^T^ | 8 | - MAQQSPAA – PMERQEDhaDhaDhaRNNLPRDhaVVGYADhbPCVI - MGSPFGSLLRCSAGA-CPRPRAAFDhaVAFRRHQAVVPCRWRRPPRADhaAGRPKKFRRA - MPATRARAPASLVELSPLPQA – RRLAAQAVVLDGWAVVLDVLEPIVPRAAADhbVADQLCDhaRQLDhbAPAHIL |
| *S. pinicola* KCTC 49008^T^ | 27 | - VTVVPSSSAQSVASTARSAGA – RVRAWFIVPRFLCVGRRGEPRPDPGDhaLAVAVVFRELVADFQDhaFARKLQDhb - MPSAAAAHPGRIVDAWLAGA – EdhbAFGKDNPAGPLFVGGGAAEAALDhbDDhaDhbDALMAFCDhaDha   PdhbGDhaYHDhaYCC |
