# Supplementary figures and images for: Genome Mining of the Genus Streptacidiphilus for Biosynthetic and Biodegradation Potential
Source: Genes (Basel). 2020 Oct 3;11(10):1166. doi: 10.3390/genes11101166 (PMC7601586; doi:10.3390/genes11101166)

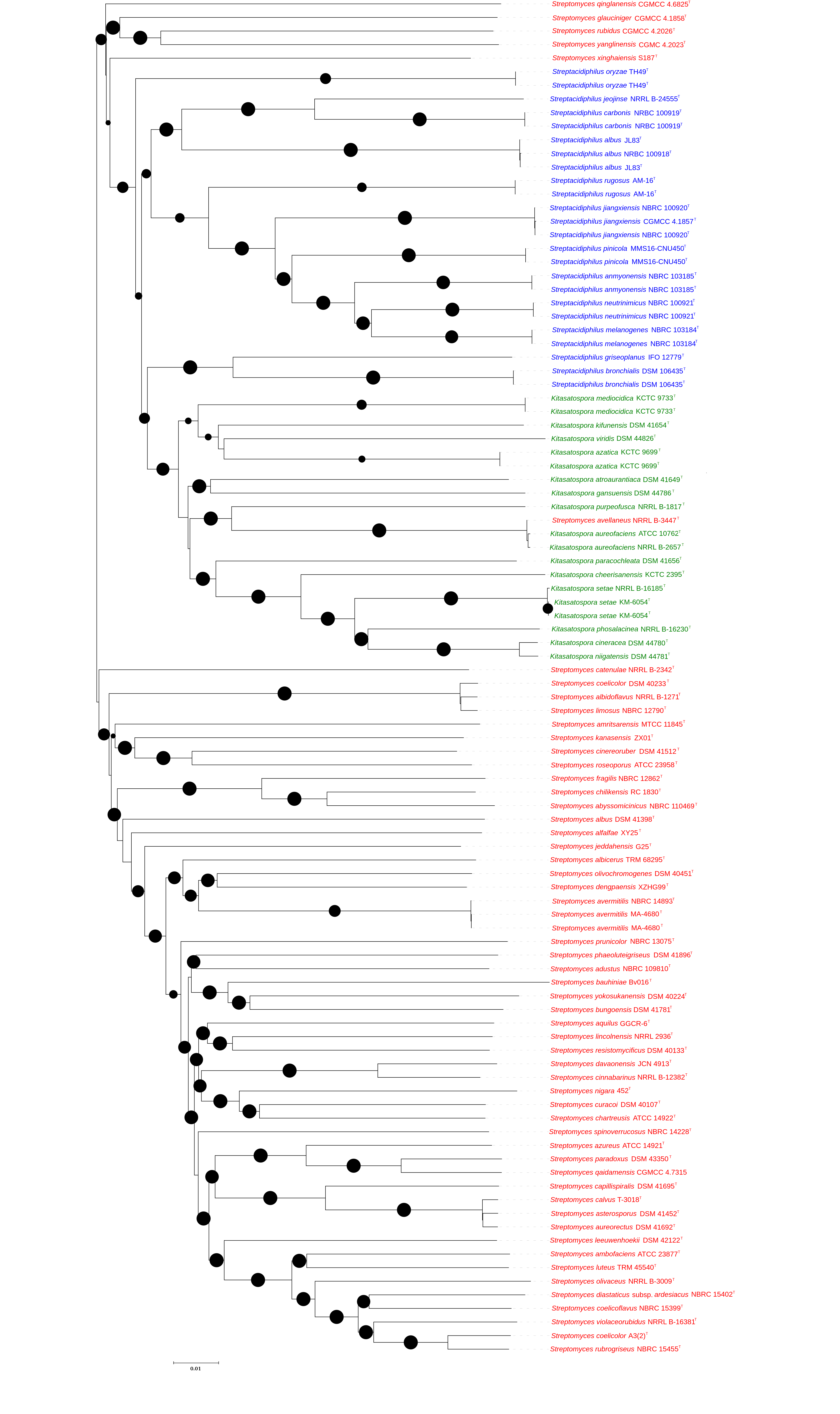

Supplement: Supplementary file 1 [file genes-11-01166-s001.zip › Figure S1-final.png]

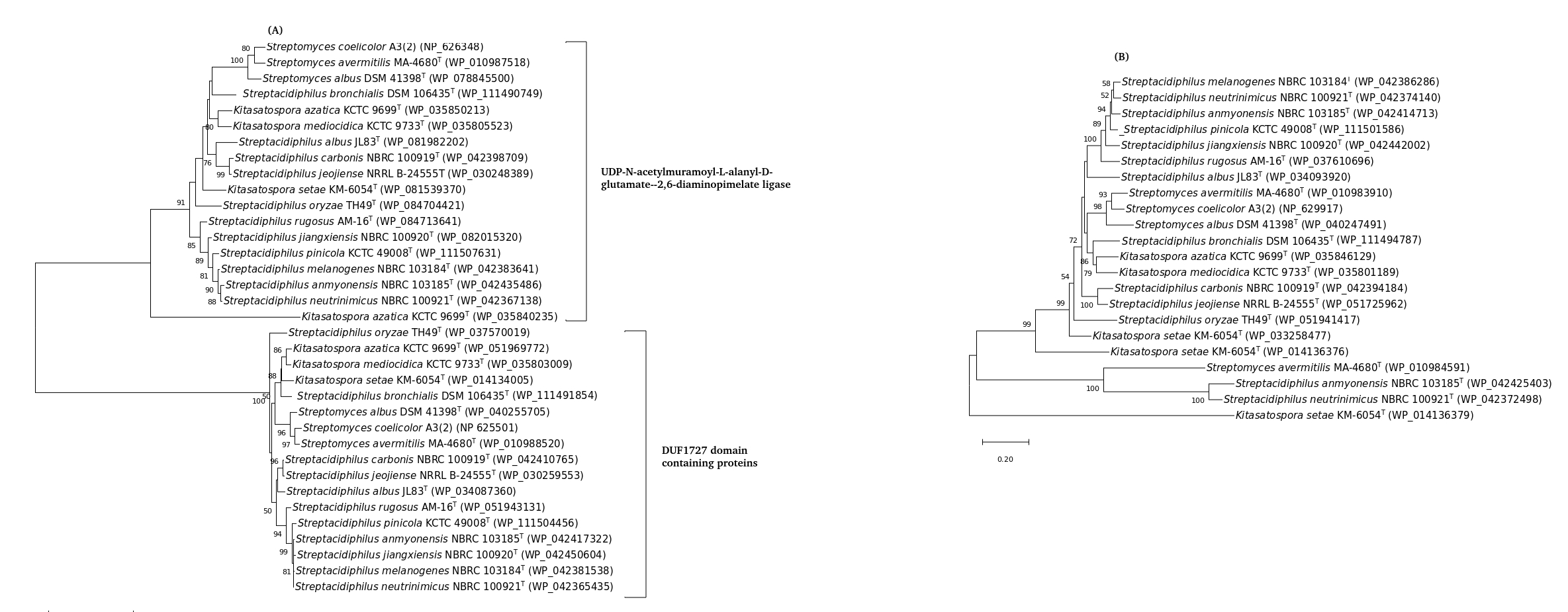

Supplement: Supplementary file 1 [file genes-11-01166-s001.zip › Figure S2-final.jpg]

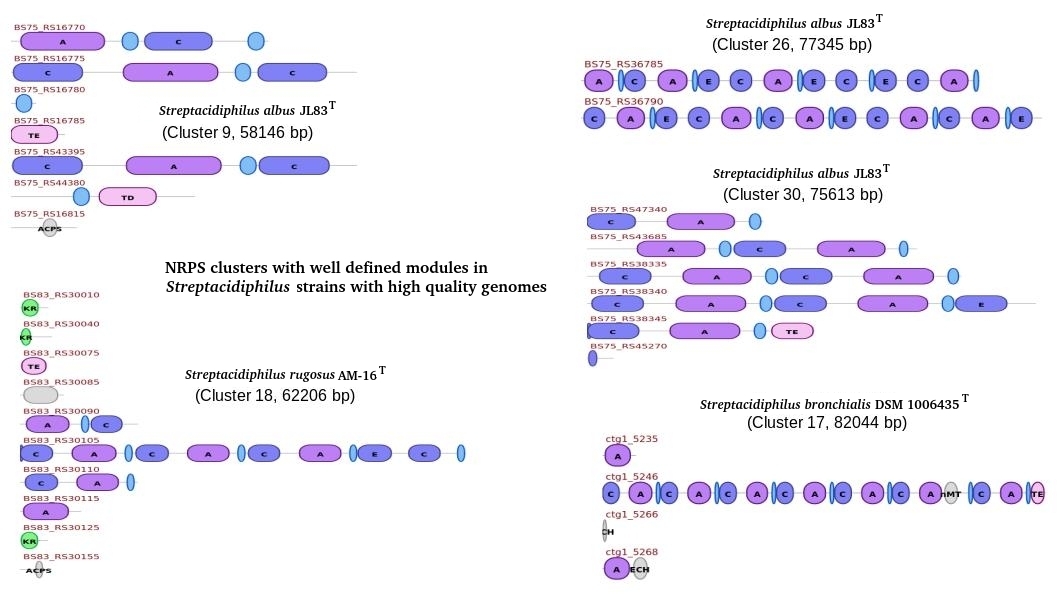

Supplement: Supplementary file 1 [file genes-11-01166-s001.zip › Figure S3-final.jpg]

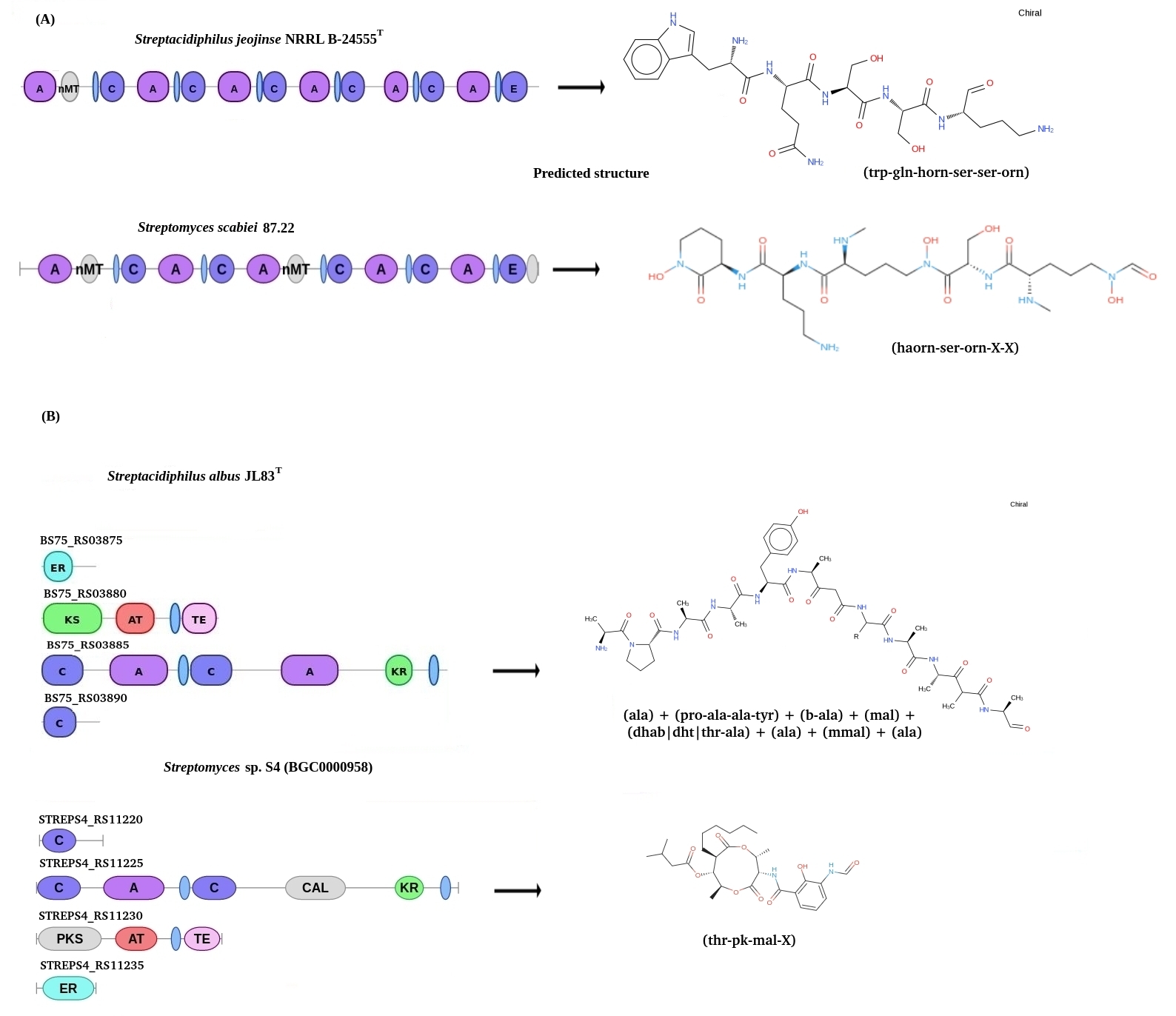

Supplement: Supplementary file 1 [file genes-11-01166-s001.zip › Figure S4-final.jpg]

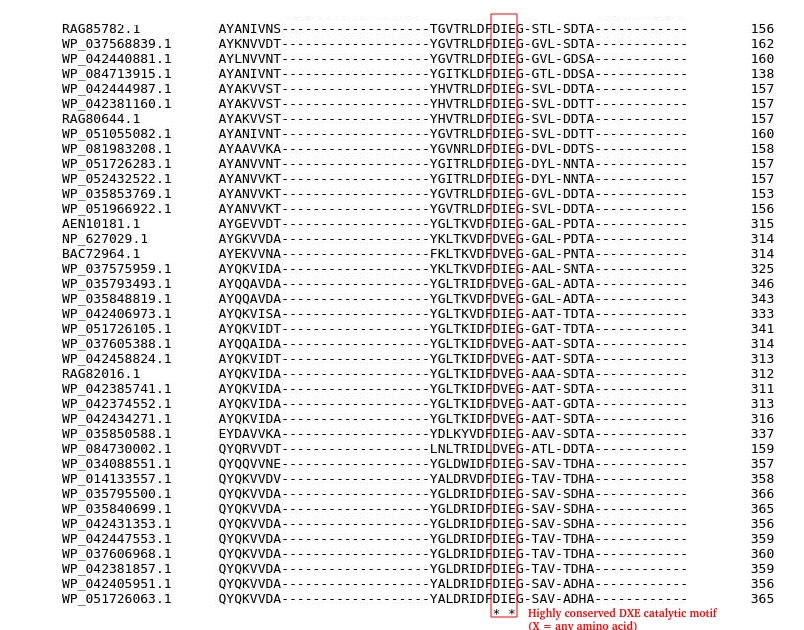

Supplement: Supplementary file 1 [file genes-11-01166-s001.zip › Figure S5-final.jpg]

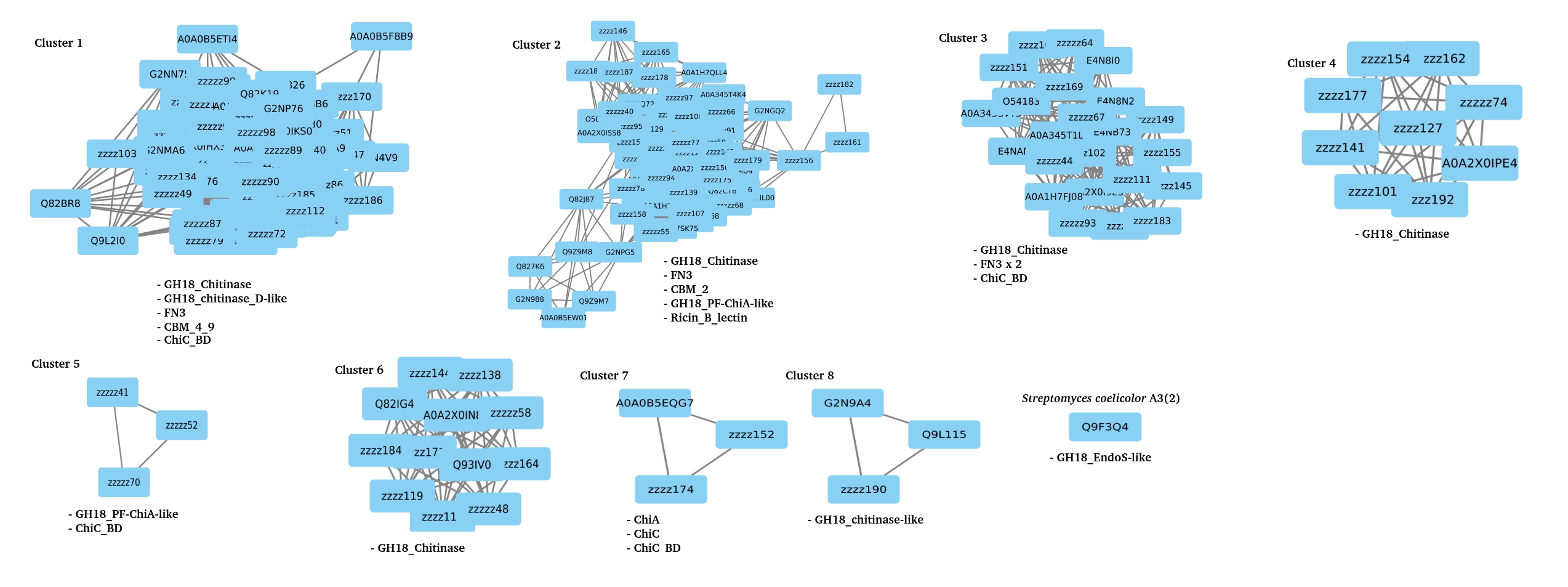

Supplement: Supplementary file 1 [file genes-11-01166-s001.zip › Figure-S6-final.jpg]
